# A hypoxia-responsive supramolecular formulation for imaging-guided photothermal therapy

**DOI:** 10.7150/thno.67036

**Published:** 2022-01-01

**Authors:** Tian-Xing Zhang, Xiaoxue Hou, Yong Kong, Fan Yang, Yu-Xin Yue, Muhammad Raza Shah, Hua-Bin Li, Fan Huang, Jianfeng Liu, Dong-Sheng Guo

**Affiliations:** 1 College of Chemistry, Key Laboratory of Functional Polymer Materials (Ministry of Education), State Key Laboratory of Elemento-Organic Chemistry, Tianjin Key Laboratory of Biosensing and Molecular Recognition, Nankai University, Tianjin 300071, China.; 2 Key Laboratory of Radiopharmacokinetics for Innovative Drugs, Chinese Academy of Medical Sciences, Institute of Radiation Medicine Chinese Academy of Medical Science & Peking Union Medical College, Tianjin 300192, China.; 3 Research Institute of Petroleum Engineering, Sinopec, Beijing 100101, China.; 4 H.E.J. Research Institute of Chemistry, International Center for Chemical and Biological Sciences, Karachi University, Karachi 74200, Pakistan.

**Keywords:** supramolecular chemistry, photothermal therapy, hypoxia, calixarene, IR780

## Abstract

Photothermal agents (PTAs) based on organic small-molecule dyes emerge as promising theranostic strategy in imaging and photothermal therapy (PTT). However, hydrophobicity, photodegradation, and low signal-to-noise ratio impede their transformation from bench to bedside. In this study, a novel supramolecular PTT formulation by a stimuli-responsive macrocyclic host is prepared to overcome these obstacles of organic small-molecule PTAs.

**Methods:** Sulfonated azocalix[4]arene (SAC4A) was synthesized as a hypoxia-responsive macrocyclic host. Taking IR780 as an example, the supramolecular nanoformulation IR780@SAC4A was constructed by grinding method, and its solubility, photostability, and photothermal conversion were evaluated. The hypoxia tumor-selective imaging and supramolecular PTT of IR780@SAC4A were further evaluated *in vitro* and *in vivo*.

**Results:** IR780@SAC4A is capable of enhancing the solubility, photostability, and photothermal conversion of IR780 significantly, which achieve this supramolecular formulation with good imaging-guided PTT efficacy *in vitro* and *in vivo*.

**Conclusions:** This study demonstrates that the supramolecular PTT strategy is a promising cancer theranostic method. Moreover, this supramolecular approach is applicative to construct kinds of supramolecular PTAs, opening a general avenue for extending smart PTT formulations.

## Introduction

Photothermal therapy (PTT) has been recognized as a clinically promising therapeutic treatment for cancer due to its lower invasive burden and high spatiotemporal selectivity 1,2. PTT relies on photothermal agents (PTAs) that can convert the absorbed light energy into heat, which causes the local temperature of the tumor issues increasing, and finally plays the role of cancer cell apoptosis inducer. Based on related literatures, the synthesized PTAs can be categorized into three types: organic small-molecule dyes, inorganic nanomaterials, and polymer agents 3,4. Due to the good biodegradability and ease of renal clearance, the organic small-molecule dye type of PTAs represent one of the great success in the field of PTT 4. Moreover, organic small-molecule PTAs exhibit great potential of easy reproducibility, controlling the preparation, and versatile synthetic modification 5. For example, IR780, a prototypic heptamethine cyanine dye, offer promising therapeutic outcomes and has attracted a lot of interest in imaging and PTT 6. However, most of organic small-molecule dyes have poor water solubility, poor photostability, and low signal-to-noise ratio 7-9, which affect their tumor imaging and therapeutic efficiency. So far, many studies have been devoted to overcoming these disadvantages by encapsulating organic dyes into various nanomaterials 5,10,11.

Supramolecular chemistry provides an alternative approach to improve the photophysical and photochemical properties of organic small-molecule dyes. Significant modulations in the photophysical and photochemical properties of PTAs can be achieved *via* the formation of supramolecular complex with macrocyclic hosts 12,13. These modulations involve p*K*_a_ shifts 14, improved solubility 14-16, fluorescence quenching or enhancement 12,17, hypsochromic or bathochromic shift of wavelengths 18,19, enhanced photochemical stability 19,20, etc. The formed supramolecular dyes are now widely utilized for applications in, but by no means limited to sensing 21,22, bioimaging 23-25, supramolecular dye lasers 26,27, organic luminescent materials 28-30, phototherapies 31-44. Moreover, as carriers for therapeutic and imaging reagents, supramolecular design owns advantages of molecular-level control of composition, dynamic reversibility, universal platform, quantitative loading, and reproducibility 21, 45-47. It is conceivable that supramolecular dyes provide enormous potential for the construction of smart PTAs based on organic small-molecule dyes.

**Scheme 1:**
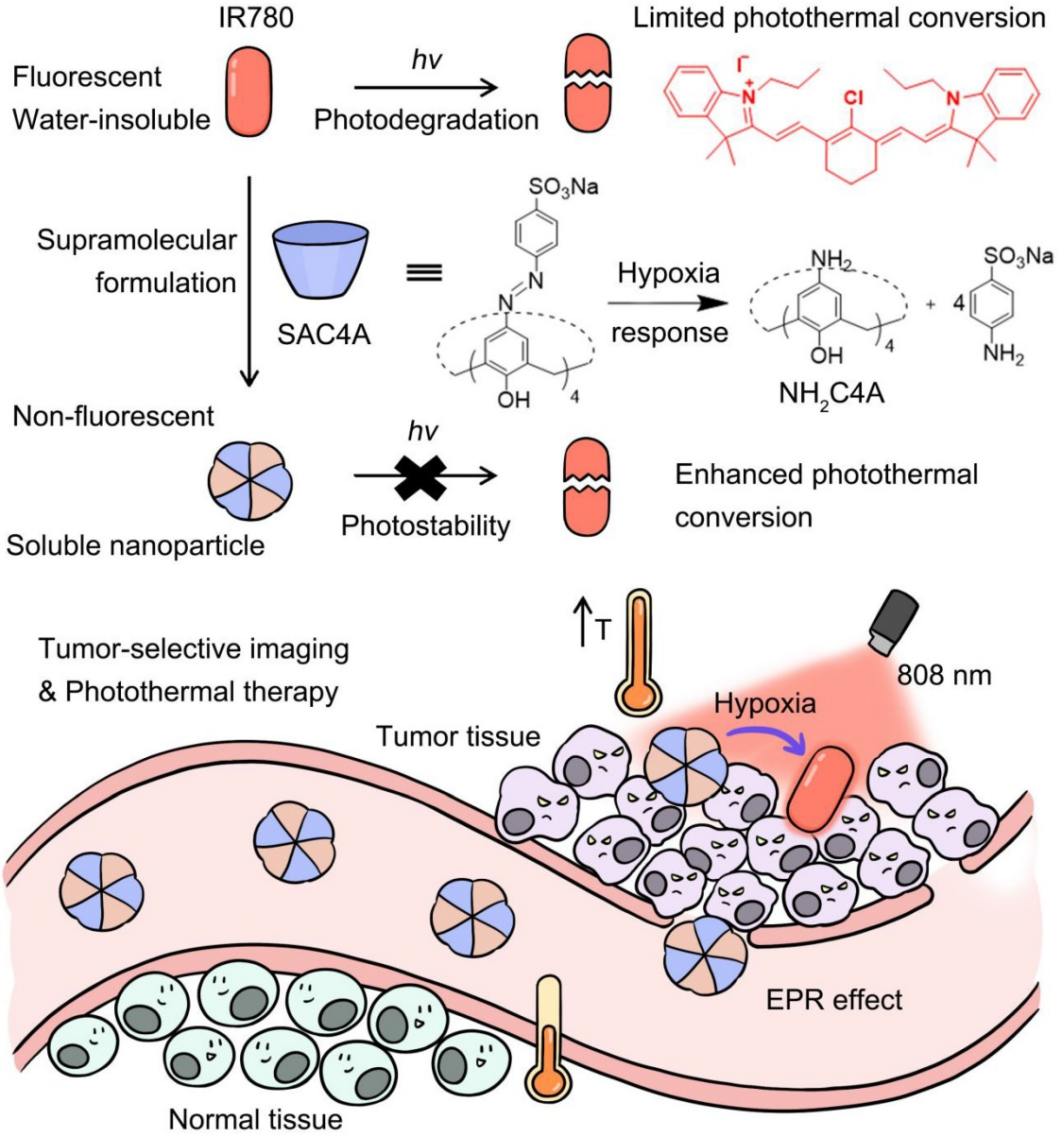
Schematic illustration of supramolecular PTA formed by the host-guest complexation between SAC4A and IR780. The encapsulation of IR780 with SAC4A gives rise to the solubility, photostability and photothermal conversion improved, but the fluorescence annihilated. Upon reaching the tumor tissue, the host responses to the hypoxia microenvironment, leading to the release of IR780 with fluorescence recovered. The supramolecular nanoformulation IR780@SAC4A therefore achieves tumor-selective imaging and PTT concurrently.

In this work, to overcome these limitations of organic small-molecule PTAs, we proposed a supramolecular PTT strategy (**Scheme 1**) by a stimuli-responsive macrocyclic host with azo bonds. Azo derivatives can be gradually reduced stepwise into aniline derivatives by various reductases 48. The encapsulation of PTA with the supramolecular host gives rise to the solubility, photostability and photothermal conversion improved, but the fluorescence annihilated. Upon reaching the lesion site, the host responses to the disease microenvironment, leading to the release of PTAs with fluorescence recovered. Consequently, disease-selective imaging and imaging-guided PTT are envisioned concurrently. As a proof of concept, IR780 was selected as an organic small-molecule PTA. Specifically, sulfonated azocalix[4]arene (SAC4A) was synthesized as a stimuli-responsive macrocyclic host. Grinding these two components generated the supramolecular formulation IR780@SAC4A with good water solubility. In the hypoxic area of the tumor, reductase is overexpressed, resulting in highly bioreductive activation 49. Therefore, when IR780@SAC4A reached the tumor site, the azo linker of SAC4A could be reduced by reductases to form aminocalix[4]arene (NH_2_C4A), leading to the dissociation of IR780 from the macrocyclic cavity. The hypoxia-responsive release of IR780 (fluorescence ON state) from IR780@SAC4A (fluorescence OFF state) implements the tumor-selective imaging. Meanwhile, both SAC4A and NH_2_C4A are able to increase the photostability and photothermal conversion of IR780, which further enhance the PTT efficacy. We verified the hypoxia tumor-selective imaging and supramolecular PTT of IR780@SAC4A *in vitro* and *in vivo*. This study not only demonstrates that the supramolecular PTT strategy is a promising approach for cancer therapy but also manifests that supramolecular dyes with generality and simplicity can serve as an ideal platform to address limitations of organic small-molecule PTAs.

## Materials and Methods

### Synthesis of SAC4A

Sulfanilic acid (1.732 g, 10 mmol) was dissolved in water (10 mL) containing sodium carbonate (0.518 g, 5 mmol) at 50-55 °C. A solution of NaNO_2_ (0.702 g, 10 mmol) in water (10 mL) was added to sulfanilic acid solution and then this mixture was added slowly to the concentrated HCl (4 mL) at 0-5 °C for 30 min and further stirred for 1.5 h at this temperature to form 4-sulfobenzenediazonium chloride salt. The obtained solution was slowly added into a solution of 25,26,27,28-tetrahydroxycalix[4]arene (C4A, 1.000 g, 2.36 mmol) and sodium acetate trihydrate (4.080 g, 30 mmol) in MeOH-DMF (26 mL, 5:8, v:v) to obtain a red suspension. The red reactant was coupled in an ice bath for 2 h more, then acidified by 150 mL of aqueous HCl (0.25%) and heated for 30 min at 60 °C to produce a reddish viscous solid. The solvent was evaporated, and the residue was treated with chloroform to obtain a precipitate. After filtration, the precipitate was dissolved by water and the insoluble solid was removed. When methanol was added, a new precipitate was formed. Then by filtration, the pure product was obtained (2.467 g, 84%). ^1^H NMR (400 MHz, DMSO-*d_6_*) *δ* 7.77 (s, 8H, calix-Ar-H), 7.69 (m, 16H, Ar-H), 4.39 and 3.66 (s, 8H, Ar-CH_2_-Ar) ppm; Mass spectrum (MALDI-TOF): [M+Na]^+^: *m/z* calcd. for (C_52_H_36_N_8_Na_5_O_16_S_4_^+^): 1271.0621, found: 1271.0618.

### Hypoxia response

The hypoxia response of SAC4A was detected by UV-Vis spectroscopy. The time-dependent absorption of SAC4A (10 μM) at 420 nm was measured in the presence of sodium dithionite (SDT, 1 mM, 100 equivalent) at 25 °C. The reduction product of SAC4A was further examined by using mass spectrometer. Besides SDT, the time-dependent absorption of SAC4A (3 μM) was measured in PBS buffer of DT-diaphorase (1 μM) and NADPH (50 μM) under hypoxic or normoxic conditions. Argon gas was bubbled into the solution for 40 minutes to create the hypoxic environment.

### Binding affinities

The direct fluorescence titration and the competitive fluorescence titration methods were used to obtain their binding constants. Rhodamine B was employed as the reporter dye. For the direct titrations, the fluorescence intensities of dye solutions were firstly measured. After sequentially adding the host solutions, the changes of fluorescence intensity and the corresponding hosts concentrations were used to determine the association constant (*K*_a_) of the host and guest. The data were fitted according to the 1:1 binding stoichiometry. The competive fluorescence titrations were taken by gradually adding competitor (IR780) to the solutions with reporter pairs whose association constants were already known. The association constants between IR780 and hosts were obtained by fitting fluorescence intensities at the emission wavelengths and cocentrations of IR780 according to the 1:1 competitive binding model.

### Solubility measurements

The excess solid of SAC4A in PBS buffer (10 mM, pH = 7.4) was shaken at 25 °C for 2 h, and the insoluble solid was removed by centrifugation at 12000 rpm for 5 min. UV-Vis spectroscopy was used to determine the concentration of SAC4A in supernatants.

Briefly, 0.006 mmol of IR780 were mixed with 1 equiv. of SAC4A in a mortar. The mixture was ground for 30 min and was collected with 6 mL PBS in a bottle. The suspension was shaken for 2 h (200 rpm, 25 °C) and then centrifuged at 10000 rpm for 10 min. Filter the supernatant with 0.22 μm water membrane and the concentration of IR780 in supernatants obtained again was determined by HPLC 50. Carried on Agilent® XDB C18 column (4.6 mm × 250 mm, 5 μm) at 30 °C, the chromatographic separation was accomplished by mobile phase consisting of acetonitrile and water (65:35) with 0.1% acetic acid in an isocratic elution. The detection wavelength was 780 nm. The flow rate of mobile phase was 1 mL min^-1^ and the injection volume was 10 μL. The solubility enhancement assay was performed in triplicate. Solubilization effect of SAC4A on IR780 is expressed by S/S_0_: S_0_ is the saturation solubility of IR780 in water (< 0.4 μg mL^-1^) 6; S is the concentration of IR780 in SAC4A solutions.

### Size and morphology evaluations

IR780@SAC4A (10 μM for IR780) in PBS was detected for the size measurement by DLS. TEM grids were prepared by evaporating 20 μL of IR780@SAC4A (10 μM for IR780) onto a copper grid.

### Photostability evaluations

2 mL of IR780, IR780@SAC4A or IR780@NH_2_C4A with the same IR780 concentration (5 μM) in the cuvette were irradiated with 808 nm wavelength laser (1 W cm^-2^) for 6 min, respectively. UV-Vis absorption tests were measured every 40 s, and the time-dependent decay of the IR780 normalized absorbance was used to indicate changes in the maximum absorption value. IR780, IR780@SAC4A or IR780@NH_2_C4A (1 mL, 50 μM for IR780, diluted by PBS) was exposed to 808 nm wavelength laser (1 W cm^-2^). Each cycle of irradiation lasted 3 min. The sample temperature of IR780, IR780@SAC4A or IR780@NH_2_C4A was measured by an infrared thermal imaging camera (Fluke, ST20 MAX). Each sample was measured for three times.

### Photothermal effects

To evaluate the photothermal effects, IR780, IR780@SAC4A or IR780@NH_2_C4A (1 mL, 20 μM for IR780, diluted by PBS 7.4) was exposed to 808 nm wavelength laser (1 W cm^-2^) with the direction of irradiation from the top to the bottom of the cuvette. The negative control PBS, SAC4A (20 μM) or NH_2_C4A (20 μM) was exposed with the same laser irradiation. The thermal images and temperatures of solution were taken by an infrared thermal imaging camera (Fluke, ST20 MAX) every minute for 15 min. The pseudo-color images were processed by SmartView software.

### Cellular uptake experiment and hypoxia imaging

4T1 cells (1 × 10^5^ cells per well) were seeded and cultured in confocal microscopic dishes. After adherence, the culture medium was replaced with fresh medium containing IR780 or IR780@SAC4A (10 μM for IR780). And then, the cells were incubated at 37 °C for different times (1, 3, 6 h) under normoxic or hypoxic conditions, respectively. Cells were washed three times with PBS buffer before imaging by an inverted fluorescence microscope.

The amount of IR780 accumulation in the cells was determined by UV-Vis spectroscopy. Cells were seeded and cultured in 10 cm dishes. After adherence, the cells were treated with IR780@SAC4A (10 μM for IR780) under normoxic and hypoxia conditions. The absorption values of cell supernatant containing IR780@SAC4A at 780 nm were measured before and after incubation with IR780@SAC4A. The IR780 content of cell supernatant before and after incubation with IR780@SAC4A were further evaluated according to the predefined concentration standard curve of IR780. Finally, intracellular IR780 content was calculated as follows:

Intracellular IR780 content = (the IR780 content of cell supernatant before incubation with IR780@SAC4A - the IR780 content of cell supernatant after incubation with IR780@SAC4A) / the number of cells.

The uptake efficiency of IR780@SAC4A was further investigated by flow cytometric analysis. 4T1 cells (2 × 10^5^ cells per well) were seeded and cultured in 6-well plates. After adherence, the culture medium was replaced with fresh medium containing IR780 or IR780@SAC4A (10 μM for IR780). And then, the cells were incubated at 37 °C for different times (1, 3, 6 h) under hypoxic conditions. After that, the medium was removed and the cells were washed with PBS several times before flow cytometric analysis.

### *In vitro* cytotoxicity assays

Briefly, cells were seeded at a density of 5 × 10^3^ cells per well into the 96-well plate. After adherence, the cells were treated with SAC4A or NH_2_C4A of different concentrations (ranging from 1, 2, 4, 8, 16 μM). And then, the cells were incubated at 37 °C for 24 h. Subsequently, the culture medium was replaced with 100 μL fresh medium and 10 μL MTT solution (5 mg mL^-1^). After incubation for 4 h at 37 °C, the medium was discarded and 150 μL of DMSO was supplemented, followed by gently shaking for 10 min. The microplate reader (Bio-Rad, iMark, USA) was utilized to record the absorbance at 570 nm. The relative cell viabilities were calculated as: cell viability = (OD_570(samples)_-OD_570(blank)_)/(OD_570(control)_-OD_570(blank)_), where OD_570(control)_, OD_570(samples)_ and OD_570(blank)_ were calculated in the absence or presence of SAC4A and in the presence of PBS, respectively. Each value was averaged from four independent experiments.

The anti-proliferation ability of IR780 and IR780@SAC4A was measured by MTT assays. 4T1 cells were cultured into the 96-well plate (5 × 10^3^ cells per well). After adherence, the cells were incubated with IR780 and IR780@SAC4A of different concentrations (ranging from 0.4, 0.8, 1.6, 3.2 μM for IR780). After incubation for 8 h, the culture medium was replaced with fresh medium. And then, the cells were received 808 nm laser irradiation (1 W cm^-2^) for 5 min, while the cells without laser irradiation were used for comparison. After incubation for 18 h, the culture medium was replaced with 100 μL fresh medium and 10 μL MTT solution (5 mg mL^-1^), followed by incubation at 37 °C for another 4 h. Thereafter, the medium was discarded and 150 μL of DMSO was supplemented, followed by gently shaking for 10 min. The absorbance at 570 nm was measured, and the relative cell viabilities were calculated as mentioned above. Each value was averaged from four independent experiments.

### *In vivo* fluorescence imaging

To establish the 4T1 tumor-bearing mouse model, 1 × 10^6^ 4T1 cancer cells were injected subcutaneously into the right chest of BALB/c nude mice at 5-6 weeks. The mice with tumor volumes at around 200 mm^3^ were randomized into two groups and intravenous injected with 100 μL of IR780 (450 μM, 1.50 mg kg^-1^) and IR780@SAC4A (450 μM for IR780, 1.50 mg kg^-1^). *In vivo* fluorescence imaging of IR780 and IR780@SAC4A were imaged by IVIS Lumina imaging system (Caliper Life Sciences, USA) at the time of 6 h, 12 h and 24 h after injection, respectively. Fluorescent images were analyzed using Living Image 3.1 (Caliper Life Sciences). For the *ex vivo* tissues distribution study, the mice were sacrificed after 24 h injection, and tumors as well as major organs (heart, liver, spleen, lung, kidney, intestines) were collected and subjected for *ex vivo* imaging. In order to quantify the signal, setting that the fluorescence of one end of dotted line in tumor sites is 100%, quantitated fluorescence intensity of the spots in the dotted line from Image J software. Distance means the length from one end of dotted line in tumor sites to other points on the line.

### *In vivo* antitumor efficacy study

To investigate the antitumor efficiency of IR780@SAC4A, 1 × 10^6^ 4T1 cancer cells were injected subcutaneously into the right flanks of the mice. The mice with tumor volumes at around 50 mm^3^ were randomized into eight groups (5 mice per group) and intravenous injected with PBS (100 μL per dose), IR780 (450 μM, 100 μL per dose, 1.50 mg kg^-1^), SAC4A (450 μM, 100 μL per dose, 2.81 mg kg^-1^) and IR780@SAC4A (450 μM for IR780, 100 μL per dose, 1.50 mg kg^-1^) with or without 808 nm laser (1 W cm^-2^) irradiation for 8 min after 24 h injection. The infrared thermal imaging camera (Fluke, ST20 MAX) were utilized to record the thermal images of mice and tumor temperatures at an interval of 30 s in the experimental process. The tumor volumes were measured every two days after irradiation over a period of 21 days. Tumors were measured by using a vernier calipers and the volume (*V*) was calculated to be *V* = *d*^2^ × *D*/2, where *d* is the shortest and the *D* is longest diameter of the tumor in mm respectively. To assess potential toxicities, mice were monitored for weight loss. Tumors were collected for H&E analysis and immunofluorescence staining. Major organs including heart, liver, spleen, lung, and kidney, were collected and stained with H&E for histopathologic analysis.

### Safety evaluations

To evaluate biosafety of SAC4A, 12 female 6-8 weeks BALB/c were randomly divided into four groups (*n* = 3), followed by intravenous injection of PBS (100 μL per dose), IR780 (450 μM, 100 μL per dose, 1.50 mg kg^-1^), SAC4A (450 μM, 100 μL per dose, 2.81 mg kg^-1^) and IR780@SAC4A (450 μM for IR780, 100 μL per dose, 1.50 mg kg^-1^) three times at the interval of one week. All mice were sacrificed on Day 21. Blood samples were collected for blood chemistry assay and blood routine assay.

## Results and Discussion

### Molecular design of macrocyclic carrier SAC4A and construction of supramolecular PTA IR780@SAC4A

Due to the large size and positive charge of IR780, it is reasonable to design and synthesize a macrocyclic host with a larger cavity and negative charge to accommodate IR780 tightly. Moreover, from the perspective of PTT, there is no need to release IR780 from the host. But from the viewpoint of tumor-selective imaging, it is necessary to annihilate the fluorescence of IR780 upon complexation with host and then release it to regenerate fluorescence within tumor. In this way, imaging-guided PTT can be realized, which can further reduce damage to the surrounding normal tissues. Accordingly, we designed the artificial macrocycle SAC4A by modifying calixarene. Calixarenes are highly modifiable, and the capability to tailor the structure of a calixarene to take on various functions makes it a representative class of macrocycle for study 51. The diazonium coupling reaction of calix[4]arene with sulfanilic acid generates SAC4A mildly with a high yield (**Scheme S1**, **Figure S1**) 52. Compared with previously reported calixarene species, SAC4A has a deeper cavitand that brings high binding ability to hydrophobic drugs of large size. In addition, the -SO_3_^-^ functional groups of sulfanilic acid give calixarene good water solubility and also work as anchoring sites that supplement the cavity binding to guests, especially the positively-charged species. More importantly, the azobenzene linkers on SAC4A are hypoxia-responsive 53,54, making tumor-targeted drug delivery feasible on account of the hypoxia character of tumor microenvironment.

**Figure 1:**
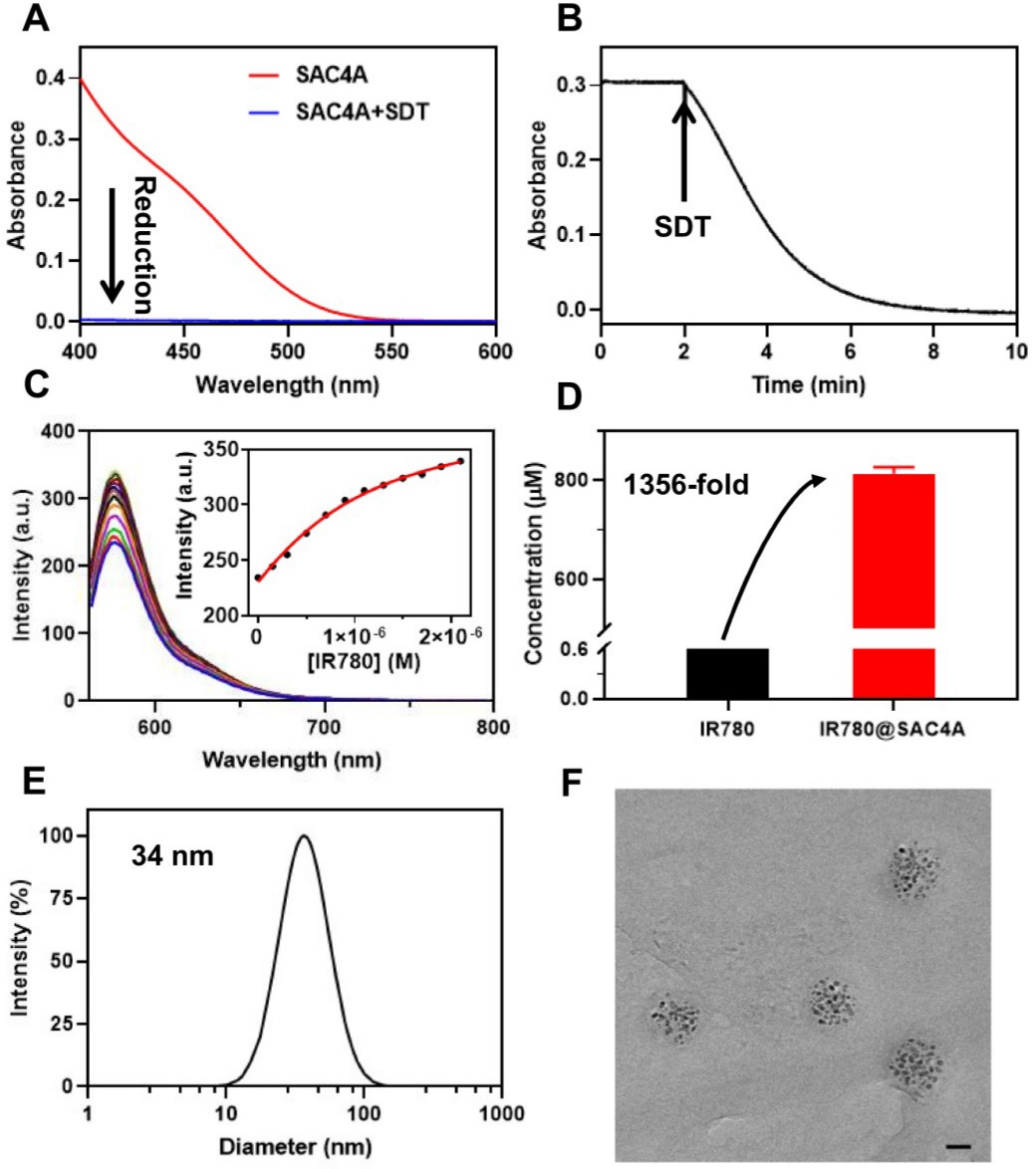
**The hypoxia response of SAC4A and characterization of IR780@SAC4A. (A)** The absorption spectra of SAC4A (10 μM) in the presence or absence of SDT (1 mM). **(B)** The absorption of SAC4A (10 μM) at 420 nm against the time in the presence of SDT (1 mM). **(C)** Competitive fluorescence titration of Rhodamine B@SAC4A (0.3/0.5 μM) with IR780 (up to 2.1 μM), *λ_ex_* = 554 nm. Inset: The associated titration curve at *λ_em_* = 575 nm was fitted according to the 1:1 competitive binding model. **(D)** The concentration of IR780 solubilized by SAC4A. The data are shown as mean ± SD (*n* = 3). **(E)** Size distribution of IR780@SAC4A. **(F)** TEM image of IR780@SAC4A. Scale bar, 20 nm. All experiments are in PBS buffer (10 mM, pH = 7.4) at 25 ºC.

We first examined the hypoxia response of SAC4A by sodium dithionite (SDT), a chemical mimetic azoreductase 55. The vanishment of the azo absorption (**Figure 1A-B**) indicates SAC4A were reduced within 8 min. We then quantified the reduction kinetics by real-time measuring of the absorbance value at 420 nm. The rate constant of 0.535 min^-1^ is obtained by the quasi-first order reaction decay model (**Figure S2**). The half-life was calculated as 78 s. The reduction product NH_2_C4A was found by mass spectrometry, indicating that four azo groups of SAC4A were completely broken (**Figure S3**). Besides SDT, the hypoxia response of SAC4A was further investigated by reduced nicotinamide adenine dinucileotide phosphate (NADPH) and DT-diaphorase, a reductase that is upregulated in tumors. SAC4A could be reduced to NH_2_C4A under hypoxic instead of normoxic conditions (**Figure S4**). The reduction property of SAC4A is quite similar to the carboxylated azocalix[4]arene (CAC4A) reported by our group before 23. We herein designed SAC4A for IR780 rather than previously adopted CAC4A, mainly because of its better water solubility. The solubility of SAC4A (**Figure S5**) in water is (191.09 ± 6.89) mg mL^-1^, much higher than that of CAC4A ((0.43 ± 0.02) mg mL^-1^) 56. The macrocyclic host with higher solubility is more conducive to solubilize organic small-molecule dyes.

To improve the solubility of IR780 by macrocyclic encapsulation, strong host-guest binding between them is prerequisite 57. We thus evaluated the encapsulation of SAC4A towards IR780. The association constant (*K*_a_), calculated from the fluorescence competitive titration data 58, was fitted as (2.1 ± 0.1) × 10^6^ M^-1^ (**Figure 1C**). Then, SAC4A solubilized IR780 was prepared by grinding method. Briefly, IR780 was vigorously ground with SAC4A powder (molar ratio of 1:1) for 30 min. The obtained mixture was firstly dispersed in PBS, and then centrifuged to remove the insoluble portion. The concentration of IR780 in the supernatant was determined by high-performance liquid chromatography, giving the concentration of (8.13 ± 0.09) × 10^-4^ M (SAC4A for 1.00 × 10^-3^ M) (**Figure 1D**). The IR780 loading efficiency in IR780@SAC4A was 30.3%, with the encapsulation efficiency about 81.3%. As far as we know, although there are many reports about solubilization of dyes by macrocycles 14, macrocyclic solubilization of IR780 has never been reported yet. Benefiting from the strong binding between SAC4A and IR780, the solubilization effect (S/S_0_) of SAC4A on IR780 is about 1356, which is superior to other carriers in solubilizing IR780 59-62.

The IR780@SAC4A complex displayed a diameter of 34 nm with a polydispersity index of 0.206 by dynamic light scattering (DLS) experiment (**Figure 1E**). The transmission electron microscopy (TEM) image revealed that IR780@SAC4A possessed a spherical morphology and the average size was measured to be 37 nm (**Figure 1F**), which was consistent with the DLS result. That is, IR780@SAC4A forms n:n nanoformulation instead of simple 1:1 host-guest complex, which may be attributed to the formation of the calixarene-induced aggregation 63,64. Such an emerging nanoformulation is capable of preferentially accumulating at tumor sites by exploiting the tumor's enhanced permeability and retention (EPR) effect 65.

### Improved photostability and photothermal conversion of IR780 upon complexation with macrocyclic hosts

**Figure 2:**
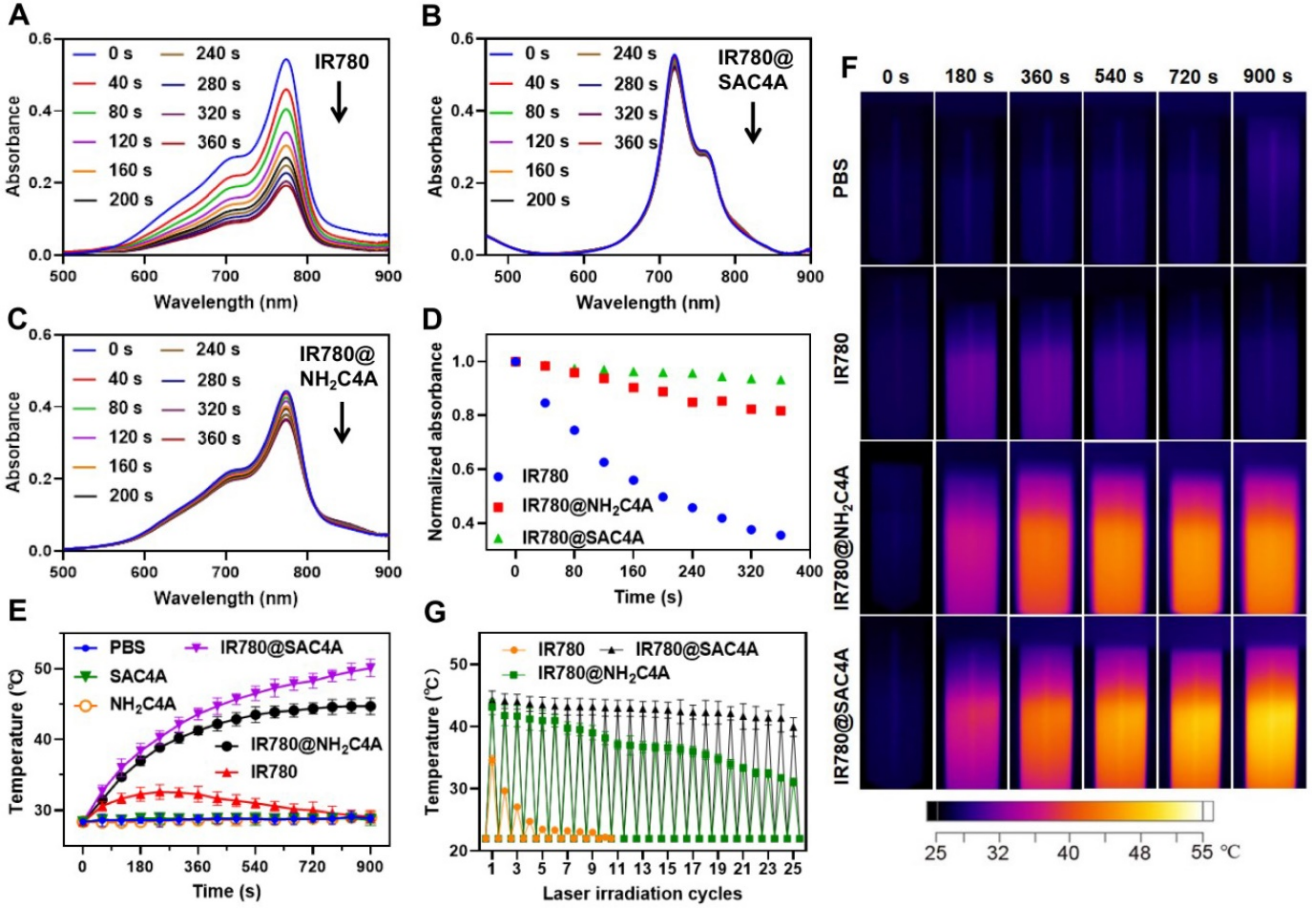
** Photostability and photothermal properties of IR780@SAC4A.** The UV-Vis absorption spectra of (**A**) IR780, (**B**) IR780@SAC4A, and (**C**) IR780@NH_2_C4A (5 μM for IR780) were recorded every 40 s for 6 min laser irradiation (808 nm, 1 W cm^-2^) at 25 ºC. **(D)** Normalized absorbance of IR780, IR780@SAC4A, and IR780@NH_2_C4A at the maximum absorption value plotted against the irradiation time. The temperature change curves (**E**) for PBS, SAC4A (20 μM), NH_2_C4A (20 μM), IR780, IR780@SAC4A, and IR780@NH_2_C4A (20 μM for IR780) solutions and thermal imaging photographs (**F**) of PBS, IR780, IR780@SAC4A, and IR780@NH_2_C4A (20 μM for IR780) solutions with irradiation. **(G)** The thermal curves of IR780, IR780@SAC4A, and IR780@NH_2_C4A (50 μM for IR780) after repeated laser irradiation. The data are shown as mean ± SD (*n* = 3). Error bars could not be shown if less than 0.4. All experiments are in PBS buffer (10 mM, pH = 7.4).

Considering that the photostability is prerequisite for satisfactory fluorescence imaging and the PTT effect, the photostability of IR780@SAC4A and IR780@NH_2_C4A was evaluated as comparison with free IR780. According to the UV-Vis absorption spectra of IR780 (**Figure 2A**), IR780@SAC4A (**Figure 2B**) and IR780@NH_2_C4A (**Figure 2C**) during the irradiation process, the photodegradation of IR780 was more obvious than IR780@SAC4A and IR780@NH_2_C4A. After 6 min laser irradiation, the normalized absorbance of IR780 markedly dropped to 35.5% of original state, while those of IR780@SAC4A and IR780@NH_2_C4A maintained 93.2% and 81.7%, respectively (**Figure 2D**). These results explicitly proved that the photostability of IR780 was observably improved by SAC4A and NH_2_C4A. The probable mechanism was that SAC4A and NH_2_C4A encapsulated IR780, which could make IR780 trapped in the complex and isolated from environment or solvent, and protect IR780 from light irradiation and oxidation 24,66.

Photothermal properties of IR780@SAC4A and IR780@NH_2_C4A were evaluated by measuring the temperature rise of solution under 808 nm near infrared laser irradiation, and PBS, SAC4A, NH_2_C4A, and free IR780 were set as control groups. As observed in **Figure 2E**, under the laser irradiation, free IR780 only caused a slight temperature increase (about 4.2 °C) in the first 4 min and the temperature then gradually decreased to the initial state. In contrast, IR780@SAC4A and IR780@NH_2_C4A displayed a significantly higher temperature increase with irradiation time and their temperature increments were approximately 21.7 °C and 16.3 °C, respectively. As the control, PBS, SAC4A and NH_2_C4A showed no appreciable temperature increase. In order to observe the temperature changes visually, the infrared thermal images (**Figure 2F**) were recorded every 3 min in a period of 15 min. For IR780@SAC4A and IR780@NH_2_C4A, the thermal imaging photographs were observed gradually deepening with increasing irradiation time while IR780 displayed a slight deepening. We further tested the photothermal effects of IR780, IR780@SAC4A, and IR780@NH_2_C4A in the experiment of repeated laser irradiation (**Figure 2G**). The photoactivity of IR780 decreased rapidly, exhibiting the poorest photostability: less than half of the initial temperature rise even just after two cycles of laser irradiation 67,68. However, after 25^th^ cycle for IR780@SAC4A and 19^th^ cycle for IR780@NH_2_C4A, their photothermal performance is still better than that of free IR780 in the first cycle. It took more than 25 cycles for IR780@SAC4A and 23 cycles for IR780@NH_2_C4A to decline to the half of the initial temperature rise. These results revealed that the photothermal properties of IR780 could be improved by encapsulating it into SAC4A and NH_2_C4A. One factor is the complexation-induced photostability enhancement. The other is the fluorescence quenching by calixarenes, which favors the pathway of nonradiative relaxation 10.

### *In vitro* hypoxia imaging and antitumor activity of IR780@SAC4A

**Figure 3:**
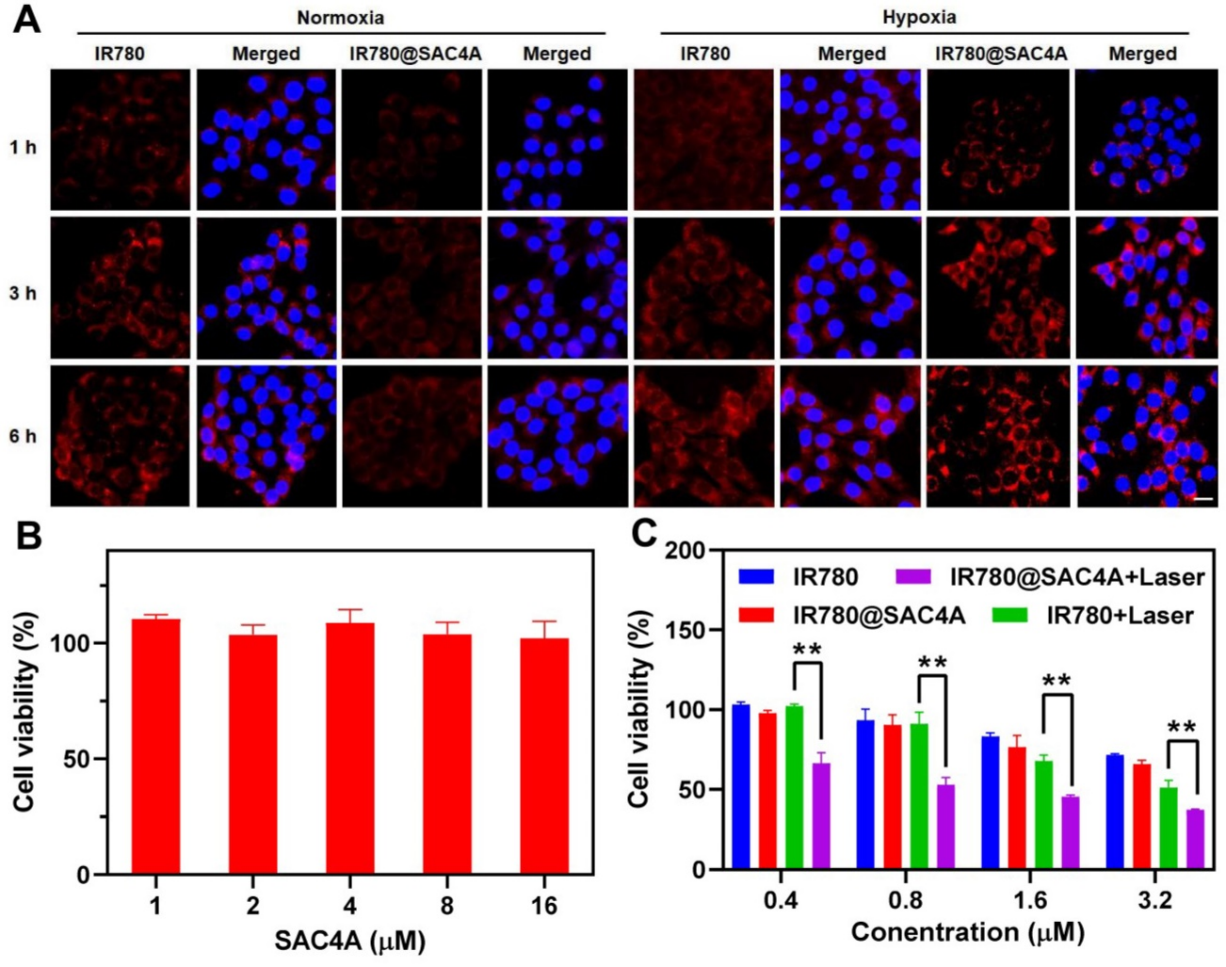
** The hypoxia response and cytotoxic actions of IR780@SAC4A. (A)** Fluorescence images of 4T1 cells after treatment with IR780 and IR780@SAC4A (10 μM for IR780) under normoxic or hypoxic conditions and stained by DAPI. Scale bar, 50 μm. **(B)** Cell viability of 4T1 cells treated with different concentrations of SAC4A for 24 h. (C) Cell viability of 4T1 cells treated with different concentrations of IR780 or IR780@SAC4A with/without laser irradiation (808 nm, 1 W cm^-2^). The data are shown as mean ± SD (*n* = 4). *P* values were calculated by Student's t test: **P < 0.01.

To verify the hypoxia imaging of IR780@SAC4A for imaging-guided PTT, we firstly investigated the host-guest complexation between NH_2_C4A and IR780. The association constant was fitted as (2.3 ± 0.3) × 10^4^ M^-1^ (**Figure S7**), about 90-times lower than IR780@SAC4A. As shown in **Figure S8**, the fluorescence of IR780 can be “switched-off” by SAC4A, and then “switched-on” again when reducing SAC4A to NH_2_C4A, which meets the requirements of hypoxia-responsive imaging 69. Then, we compared the fluorescence imaging ability of IR780@SAC4A in living cells under hypoxic and normoxic conditions. Much stronger fluorescence was observed in treated cells under hypoxic conditions than that under normoxic conditions (**Figure 3A**). The fluorescence enhancement of IR780@SAC4A may come from either the hypoxic response or the different cellular uptake under normoxic and hypoxic conditions. We tested the cellular uptake of IR780@SAC4A under hypoxic and normoxic conditions. As shown in **Figure S9**, no appreciable difference about IR780 concentration in treated cells under hypoxic and normoxic conditions was observed, indicating that hypoxia leads to negligible effect on cellular uptake of IR780@SAC4A as comparison with normoxia. Therefore, the fluorescence enhancement of IR780@SAC4A is due to the hypoxic response, suggesting that IR780@SAC4A possessed reliable hypoxia-selective imaging for tumors. Furthermore, the time-dependent cellular uptake of free IR780 and IR780@SAC4A was investigated under hypoxic conditions. The fluorescence in cells incubated with IR780@SAC4A was stronger than that of IR780 at same incubation time, which indicated that SAC4A could favorably accelerate the delivery of IR780 into cells. We further investigated the uptake efficiency of IR780@SAC4A in hypoxic cells by flow cytometry. The fluorescence intensity of cells incubated with IR780@SAC4A was remarkable stronger than that of IR780 (**Figure S10**), which was highly consistent with the results of fluorescence imaging of cellular uptake.

The *in vitro* antiproliferation effects of IR780@SAC4A towards 4T1 cells were estimated by MTT assay. We first evaluated the cytotoxicity of SAC4A (**Figure 3B**) and NH_2_C4A (**Figure S11**) at concentrations ranging from 1 to 16 μM, and the results showed that SAC4A and NH_2_C4A displayed negligible cytotoxicity to 4T1 cells. When treated 4T1 cells with free IR780 or IR780@SAC4A in the absence of irradiation, the cell viability reduced in similar degree (**Figure 3C**). However, IR780@SAC4A with laser irradiation dramatically deceased cell viability with increasing concentrations and its decline degree was stronger than that of free IR780 under laser irradiation, which indicated that IR780@SAC4A possessed superior photothermal toxicity than free IR780. The enhanced cell toxicity of IR780@SAC4A may be attributed to enhanced cellular uptake and improved photostability as compared to the free dye.

### *In vivo* tumor imaging and PTT of IR780@SAC4A

**Figure 4:**
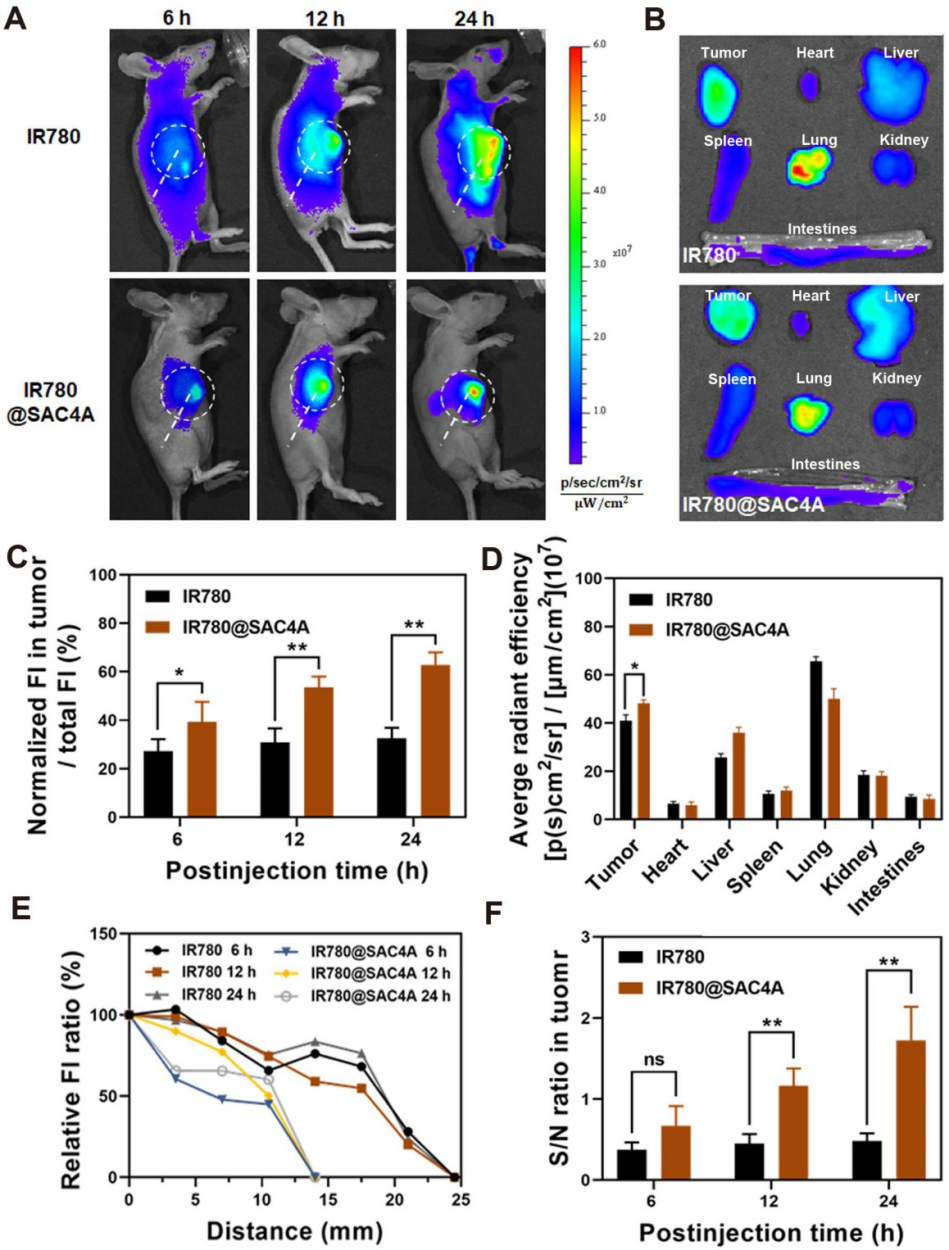
***In vivo* fluorescence imaging of IR780@SAC4A. (A)** *In vivo* fluorescence images of 4T1-bearing mice after 6, 12 and 24 h intravenous injection of IR780 and IR780@SAC4A. Tumor tissues were circled by white dotted line. **(B)** *Ex vivo* fluorescence images of dissected major organs (heart, liver, spleen, lung, kidney, intestines) and tumor after injection of IR780 and IR780@SAC4A at 24 h. **(C)** Quantitative analysis of the ratio of fluorescence intensity in tumor and whole body after injection with IR780 and IR780@SAC4A at 6, 12 and 24 h. **(D)** Quantitative analysis of fluorescence intensity of IR780 and IR780@SAC4A in major organs and tumor based on *ex vivo* images from (B). **(E)** The fluorescence intensity changes of the distance from tumor to the normal tissues. **(F)** The signal to noise ratio (S/N) of fluorescence between tumor and surrounding tissues. The data are shown as mean ± SD (*n* = 3). *P* values were calculated by Student's t test: *P < 0.05, **P < 0.01. ns represents “no significant difference”.

We then investigated the capability of IR780@SAC4A for tumor imaging *in vivo*. 4T1 tumor-bearing mice were intravenously injected with free IR780 or IR780@SAC4A, respectively, followed by recording the intrinsic fluorescence of IR780 at different time points. The fluorescence intensity in tumor of mice administrated with IR780@SAC4A gradually increased within 24 h postinjection and the signals were only concentrated near the tumor tissue (**Figure 4A**). In contrast, mice treated with free IR780 showed whole body fluorescence distribution and much weaker fluorescence intensity in tumor. Meanwhile, we quantitatively analyzed the ratio of fluorescence intensity in tumor and whole body (**Figure 4C**), and the ratio for IR780@SAC4A group remarkably increased with time while free IR780 group changed little. These results revealed that SAC4A could effectively improve the tumor accumulation of IR780 due to the EPR effect and hypoxia-responsive release. The *ex vivo* imaging and followed quantitative analysis (**Figure 4B** and **4D**) at 24 h postinjection further supported the above results. **Figure 4B** and **4D** also showed that there were fluorescence signals in the lungs of mice treated with IR780. This phenomenon might be that the agglomeration of hydrophobic IR780 dye in physiological environment were entrapped in the lungs of mice 70*.* And strong fluorescence signals in the lungs were also observed in mice treated with IR780@SAC4A, which was in accordance with the results of previous reference that IR780-loaded nanoparticles could be mechanically retained in lung capillaries 71. Moreover, it was worth noting that the distance from tumor with the distinct fluorescence intensity to the normal tissue that could not detect the fluorescence signal was clearly shorter for IR780@SAC4A than that of IR780 (**Figure 4E**), indicating the more precise imaging of IR780@SAC4A than free IR780. Addtionally, IR780@SAC4A significantly enhanced the signal to noise ratio (S/N) of tumor to surrounding tissues compared to free IR780 (**Figure 4F**). Therefore, it is confident to deduce that IR780@SAC4A has potential applications in tumor diagnosis and imaging-guided PTT.

**Figure 5:**
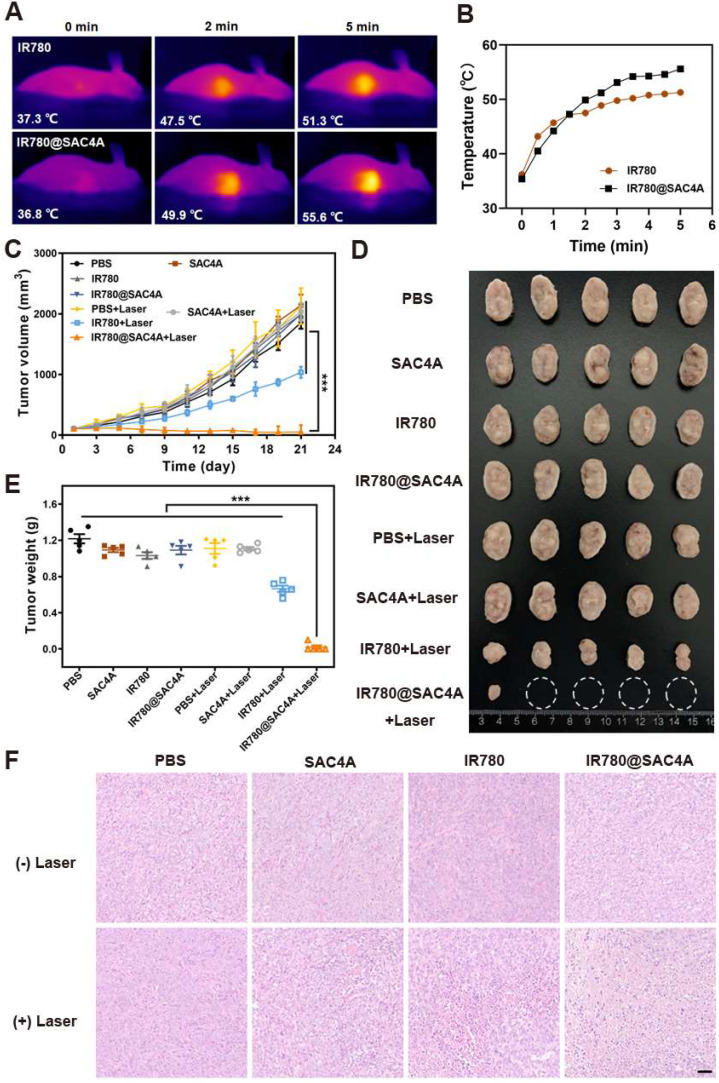
***In vivo* PTT of IR780@SAC4A. (A)** Thermal images of 4T1 tumor-bearing mice received intravenous injection of free IR780 or IR780@SAC4A upon irradiation with 808 nm laser at different time (1.0 W cm^-2^). **(B)** Changes in temperature of tumors upon 808 nm laser irradiation. **(C)** Tumor volume growth curves of mice treated with different interventions. **(D)** Images of dissected tumors in various groups. White circles indicate the eliminated tumors. **(E)** Weights of tumors dissected from mice in different groups after 21 days of treatment. **(F)** H&E staining of tumor slices after different treatments. Scale bar, 50 μm. The data are shown as mean ± SD (*n* = 5). *P* values were calculated by Student's t test: ***P < 0.001.

The significant *in vitro* therapeutic effect and *in vivo* selective lighting of tumor tissues by IR780@SAC4A imply its potentially outstanding *in vivo* PTT outcome. We first employed an infrared camera to monitor the changes of temperature in tumor region at different times. Free IR780 and IR780@SAC4A were intravenously injected into 4T1 tumor-bearing mice respectively, followed by irradiation with the 808 nm laser (1 W cm^-2^) at 24 h postinjection. As shown in **Figure 5A-B**, the increasing temperature in tumor of mice administrated with free IR780 and IR780@SAC4A in pace with prolongation of irradiation time. More importantly, the elevated tumor temperature of IR780@SAC4A groups was higher than that of free IR780. The antitumor activities of IR780@SAC4A were then investigated on 4T1 tumor-bearing BALB/c mice model. The mice were randomly divided into 8 groups (*n* = 5 per group). Then, the mice were irradiated with/without 808 nm laser (1 W cm^-2^) for 8 min at 24 h postinjection of different formulations, followed by measuring the tumor volumes every two days over 21 days. The IR780@SAC4A group with laser irradiation treatment exhibited the most obvious tumor growth suppression effect among all the interventions (**Figure 5C**). The image of tumor dissected from mice and comparison of tumor weight further validated the best *in vivo* antitumor effect of IR780@SAC4A (**Figure 5D**-**E**). To further illustrate the therapeutic efficacy, hematoxylin-eosin (H&E) staining and terminal deoxynucleotidyl transferase dUTP nick end labeling (TUNEL) assay were conducted to assess the pathology changes and apoptosis degree in tumors, respectively. As shown in **Figure 5F**, extensive morphology changes and condensed nuclei were clearly observed in tumor dissected by the mice treated with IR780@SAC4A+Laser group, whereas inappreciable apoptosis could be observed from other groups. Analogous to the result of H&E staining, more significant positive TUNEL signals were observed in tumor tissues from mice treated with IR780@SAC4A+Laser, further confirming the enhanced PTT efficiency with assist of IR780@SAC4A (**Figure S12A**). Immunofluorescence Ki67 staining was then conducted to evaluate *in vivo* antiproliferation capacity after different treatment. As shown in **Figure S12B**, IR780@SAC4A+Laser group resulted in more distinct reduced in Ki67 positive tumor cells compared with other treatments, further indicating that IR780@SAC4A could act as an excellent PTA for enhancing tumor PTT efficiency.

### Biosafety of SAC4A and IR780@SAC4A

Biosafety, as a critical concern for developing biomedicine, was evaluated to assess potential of our strategy in clinical application. As shown in **Figure S13**, no apparent variation of the body weight in various groups were observed, indicating the negligible side effects of SAC4A and IR780@SAC4A. Furthermore, major organs of mice after treatment were collected and stained by H&E for histopathologic analysis. There were no obvious tissue lesions in main organs, corresponding to the insignificant systemic toxicity of SAC4A and IR780@SAC4A (**Figure S14**). The blood biochemistry and blood routine assays were carried out to further confirm the innocuity of SAC4A and IR780@SAC4A (**Figure S15**). Compared with PBS treated mice, the levels of lactate dehydrogenase (LDH) and creatinine (CRE) in IR780 treated mice were significantly increased, indicating that IR780 has certain toxicity 6,72. In contrast, there was no evident elevation of serum enzymes in IR780@SAC4A group, and SAC4A reduced the toxic and side effects of IR780. Therefore, SAC4A is expected to be a supramolecular carrier for organic PTAs due to its good biological safety.

## Conclusion

In summary, we developed a novel supramolecular PTT formulation by host-guest complexation of SAC4A with IR780, which not only address the issues of organic small-molecule PTAs, including solubility, photostability and photothermal conversion, but also achieves imaging-guided PTT. This supramolecular formulation is easily prepared by grinding two components, where the water solubility of IR780 was dramatically improved. Upon reduction of SAC4A to NH_2_C4A in hypoxia microenvironment, IR780 was released with fluorescence recovered owing to the decreased binding affinity of NH_2_C4A than SAC4A, giving rise to the tumor-selective imaging with a high signal-to-background ratio. Both SAC4A and NH_2_C4A are capable of enhancing the photostability and photothermal conversion of IR780 significantly. Therefore, whether SAC4A is reduced to NH_2_C4A or not, released and unreleased IR780 can contribute to PTT, which insure this supramolecular formulation with good PTT efficacy *in vitro* and *in vivo*. Taking the tumor-selective imaging and high PTT efficacy into account concurrently, the present supramolecular PTT formulation fulfils highly efficient tumor ablation as well as reduced damage to surrounding normal tissues. To be envisaged, this supramolecular approach is adaptative to construct abundant supramolecular PTAs by randomly incorporating hosts and dyes as long as compatibility permits, opening a general avenue for forming smart PTT systems.

## Abbreviations

PTAs: photothermal agents
PTT: photothermal therapy
SAC4A: sulfonated azocalix[4]arene
DMF: N,N-dimethylformamide
DMSO: dimethyl sulfoxide
NH_2_C4A: aminocalix[4]arene
SDT: sodium dithionite
NADPH: reduced nicotinamide adenine dinucileotide phosphate
PBS: phosphate buffered saline
MTT: methyl thiazolyl tetrazolium
CAC4A: carboxylated azocalix[4]arene
HPLC: high-performance liquid chromatography
DLS: dynamic light scattering
TEM: transmission electron microscopy
UV-Vis: ultraviolet visible
EPR: tumor's enhanced permeability and retention effect
H&E: hematoxylin-eosin
TUNEL: terminal deoxynucleotidyl transferase dUTP nick end labeling
LDH: dehydrogenase
CRE: creatinine

## Supplementary Material

Supplementary materials and methods, scheme, figures.
