# Supplementary material for: A hypoxia-responsive supramolecular formulation for imaging-guided photothermal therapy
Source: Theranostics. 2022 Jan 1;12(1):396–409. doi: 10.7150/thno.67036 (PMC8690909; doi:10.7150/thno.67036)
Supplement: Supplementary file 1 — Supplementary materials and methods, scheme, figures. [file thnov12p0396s1.pdf]

Supplementary Material

for

A hypoxia-responsive supramolecular formulation for imaging-guided photothermal therapy

Tian-Xing Zhang[1,#], Xiaoxue Hou[2,#], Yong Kong[3], Fan Yang[3], Yu-Xin Yue[1], Muhammad Raza Shah[4], Hua-Bin Li[1], Fan Huang[2,✉], Jianfeng Liu[2,✉], Dong-Sheng Guo[1,✉]

1. College of Chemistry, Key Laboratory of Functional Polymer Materials (Ministry of Education), State Key Laboratory of Elemento-Organic Chemistry, Tianjin Key Laboratory of Biosensing and Molecular Recognition, Nankai University, Tianjin 300071, China
2. Key Laboratory of Radiopharmacokinetics for Innovative Drugs, Chinese Academy of Medical Sciences, Institute of Radiation Medicine Chinese Academy of Medical Science & Peking Union Medical College, Tianjin 300192, China
3. Research Institute of Petroleum Engineering, Sinopec, Beijing 100101, China
4. H.E.J. Research Institute of Chemistry, International Center for Chemical and Biological Sciences, Karachi University, Karachi 74200, Pakistan

# These authors contributed equally to this work.

✉ Corresponding author: Fan Huang, Prof., E-mail: huangfan@irm-cams.ac.cn. Jianfeng Liu, Prof., E-mail: liujianfeng@irm-cams.ac.cn. Dong-Sheng Guo, Prof., E-mail: dshguo@nankai.edu.cn.

Supplementary materials and methods

Materials. All the reagents and solvents were commercially available and used as received unless otherwise specified purification. IR780 and the phosphate buffered saline (PBS, pH = 7.4, 10 mM) were purchased from Macklin. Sodium dithionite (SDT) was obtained from J&K Chemical. 5,11,17,23-Tetraamino-25,26,27,28-tetrahydroxycalix[4]arene (NH_2C4A) was synthesized referring to our previous literature [1]. Rhodamine B, sulfanilic acid, and sodium nitrite were purchased from Aladdin. Dihydronicotinamide adenine dinucleotide phosphate tetrasodium salt (NADPH) was purchased from Ark. DT-diaphorase was purchased from Sigma-Aldrich. Fetal bovine serum (FBS) and Dulbecco's modified eagle medium (DMEM) were purchased from Thermo Fisher Scientific. 4'6-diamidino-2-phenylindole (DAPI) and hematoxylin-eosin (H&E) staining kit were obtained from Solarbio. Penicillin streptomycin sol was purchased from Gibco. 3-(4,5-Dimethylthiazol-2-yl)-2,5-diphenyl tetrazolium bromide (MTT) was purchased from Amresco. All antibodies for immunofluorescence staining were purchased from Biolegend and in situ terminal deoxynucleotidyl transferase dUTP nick end labeling (TUNEL) kits were purchased from Roche.

Apparatus. NMR data were recorded on a Bruker AV400 spectrometer. UV−Vis spectra were recorded in a quartz cell (light path 10 mm) on a Cary 100 UV−Vis spectrophotometer equipped with a Cary dual cell peltier accessory. Steady-state fluorescence measurements were recorded in a conventional quartz cell (light path 10 mm) on a Cary Eclipse equipped with a Cary single-cell peltier accessory. Fluorescence microscopy images were observed by an inverted fluorescence microscope (Leica, DMI 6000B). Mass spectra were performed on a Shimadzu LCMS-2020 and an Autoflex III TOF/TOF200 (MALDI-TOF). High-performance liquid chromatography (HPLC) system (Waters, Milford, MA, USA) was employed to perform chromatographic analysis. The sample solutions for dynamic light scattering (DLS) measurements were examined on a laser light scattering spectrometer (NanoBrook 173plus). The TEM sample was examined by a high-resolution TEM (Tecnai G2 F20 microscope, FEI) equipped with a CCD camera (Orius 832, Gatan). All photothermal experiments were conducted by irradiation with a 808 nm wavelength laser (Laserwave, LI-P20W). The temperatures and thermal imaging photographs of the solutions were obtained by an infrared thermal imaging camera (Fluke, ST20 MAX). Tissue sections were observed and photographed with an inverted fluorescence microscope (Leica, DMI 6000B).

Data analysis of fluorescence titrations. The fitting of data from direct host-guest titrations

and competitive titrations were performed in a nonlinear manner [2], and the fitting modules were downloaded from the website of Prof. Nau's group (http://www.jacobs-university.de/ses/wnau) under the column of "Fitting Functions". It was introduced in detail as following.

For analyzing the host-guest fluorescence titrations as described by equation 1, we considered that a guest (G) formed a 1:1 host-guest complex with a host (H) at an association constant (K_a), which satisfied the respective law of mass action relating to the equilibrium concentrations of free host, [H], free guest, [G], and host-guest complex [HG]. Also, the relationship between the total concentrations of host, $[H]_0$, and guest, $[G]_0$, and their equilibrium concentrations were introduced by the law of mass conservation (equation 2). Here, $[G]_0$ was the initial concentration of guest as a known experimental parameter, which was kept constant in the titration process. Furthermore, equation 1 and 2-1 were employed to deduce equation 3.

When the fluorescence titrations were performed, the intensity of fluorescence (F_C) corresponded to the combined intensity of the guest and the host-guest complex, which were described by their molar fractions (equation 4). Both F_{HG} and F_G were the known experimental parameters, in which F_{HG} was the fluorescence intensity when all guests were complexed and F_G when they were uncomplexed. The equation 5 deduced by equation 2-2, 3 and 4, explained the relationship between K_a and variables ($[H]_0$) in fluorescence titrations [3]. In the light of equation 5, K_a was obtained by fitting the data of fluorescence intensity and total host concentration.

$$[H]+[G] \overset{K_a}{\rightleftharpoons} [HG]$$

$$K_a = \frac{[HG]}{[H][G]} \tag{1}$$

$$[G] = [G]_0 - [HG] \tag{2-1}$$

$$[H] = [H]_0 - [HG] \tag{2-2}$$

$$[HG] = \frac{K_a[H][G]_0}{1+K_a[H]} \tag{3}$$

$$F_C = \frac{[HG]}{[G]_0} F_{HG} + \frac{[G]}{[G]_0} F_G \tag{4}$$

$$F_C = F_{HG} + (F_G - F_{HG}) \frac{([G]_0 - [H]_0 - 1/K_a) - \sqrt{([G]_0 - [H]_0 - 1/K_a)^2 + 4[H]_0[G]_0}}{2[G]_0} \tag{5}$$

For the analysis of the competitive titrations (equation 6), we also considered a competitor

(drug) that could bind to a host in a 1:1 stoichiometry at an association constant (K_D). Free host [H], free competitor [D] and host-drug complex [HD] obeyed the respective law of mass action referring to the equilibrium concentrations. Also, $[H]_0$ and the total concentrations of drug $[D]_0$, and their equilibrium concentrations satisfied the law of mass conservation (equation 7).

In the course of the titration, the fluorescence intensity (F_D) was expressed as a linear combination of F_{HG} and F_G, weighted by their molar fractions on the basis of equation 8. Through a 1:1 host-guest binding model, F_{HG} was further denoted by the (initial) experimental fluorescence intensity in the absence of drug. Substituting equation 3 into equation 8 gave equation 9, with the concentration of uncomplexed host as an unknown parameter, [H], which was numerically solved by a cubic equation (equation 10) with Newton-Raphson algorithm [4,5]. In addition, equation 10 was deduced by combining equation 3, 6, 7-1 and 7-2. For fitting, the fluorescence intensity was plotted against $[D]_0$ based on equation 9 in a program.

$$[H]+[D] \underset{}{\overset{K_D}{\rightleftharpoons}} [HD]$$

$$K_D = \frac{[HD]}{[H][D]} \quad (6)$$

$$[H] = [H]_0 - [HG] - [HD] \quad (7\text{-}1)$$

$$[D] = [D]_0 - [HD] \quad (7\text{-}2)$$

$$F_D = \frac{[HG]}{[G]_0} F_{HG} + \frac{[G]}{[G]_0} F_G \quad (8)$$

$$F_D = F_G + (F_{HG} - F_G)\frac{K_a[H]}{1+K_a[H]} \quad (9)$$

$$\begin{aligned} &0 = A[H]^3 + B[H]^2 + C[H] - D, \\ &\text{where} \\ &A = K_a K_D \\ &B = K_a + K_D + K_a K_D([G]_0 + [D]_0 - [H]_0) \\ &C = K_D([D]_0 - [H]_0) + K_a([G]_0 - [H]_0) + 1 \\ &D = -[H]_0 \end{aligned} \quad (10)$$

Cell culture. 4T1 cell line was purchased from Cell Bank of the Chinese Academy of Sciences (Shanghai, China). DMEM with 10% FBS, and 1% penicillin and streptomycin were used as the cell culture medium. Cells were incubated in a 5% CO_2 humidified incubator at 37 °C. A humidified atmosphere containing 5% CO_2 was used as normoxic cell culture environment. The hypoxic cell culture environment was adjusted by purging gas mixture

(94% N_2, 5% CO_2, 1% O_2).

Mice. For animals and tumor model, female BALB/c mice at 6-8 weeks and female nude BALB/c nude mice at 5-6 weeks were purchased from Vital River Laboratory Animal Technology (Beijing, China). All animal procedures were performed in accordance with the Guidelines for the Care and Use of Laboratory Animals of Peking Union Medical College and experiments were approved by the Animal Experiments and Ethics Review Committee of the Institute of Radiation Medicine, Chinese Academy of Medical Sciences.

Immunofluorescence staining. Immunofluorescence staining was performed on frozen tumor sections. Briefly, harvested tumors were first placed in 4% paraformaldehyde for 48 h at 4 °C, and then transferred to 15% then 30% sucrose solution (w/w) for dehydration. The tumors were embedded in optimal cutting medium, and frozen slices were made on a cryostat microtome. Immunofluorescence staining was performed by rinsing with PBS, permeabilization, followed by blocking in 5% BSA at room temperature for 1 h, and then stained with primary antibodies-Ki67, overnight at 4 °C following the manufacturer's instructions. Following the addition of fluorescently labelled secondary antibodies, the slides were analyzed with fluorescence microscopy images. All antibodies used in the experiments were diluted 200 times. For the terminal deoxynucleotidyl transferased dUTP nick end labeling (TUNEL) apoptosis staining, the fixed tumour sections were determined following manual instruction of in situ Cell Death Detection Kit (Roche Applied Science).

Statistical analysis. All results are shown as mean ± standard deviation (SD). Data were analyzed by Student's t-test. *P* values less than 0.05 were considered statistically significant.

Supplementary figures

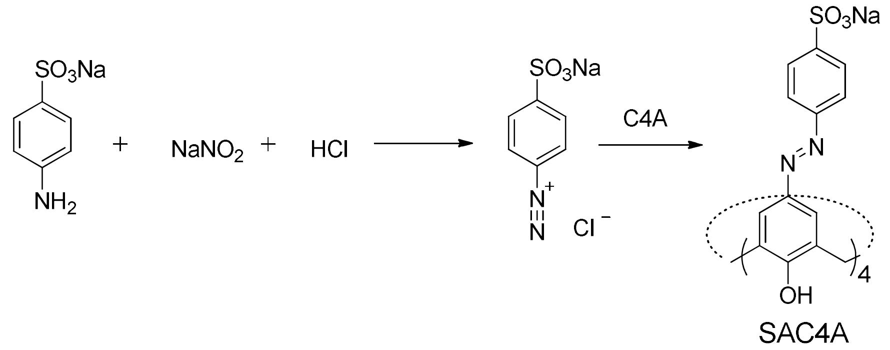

Scheme S1. The synthetic route of SAC4A.

a

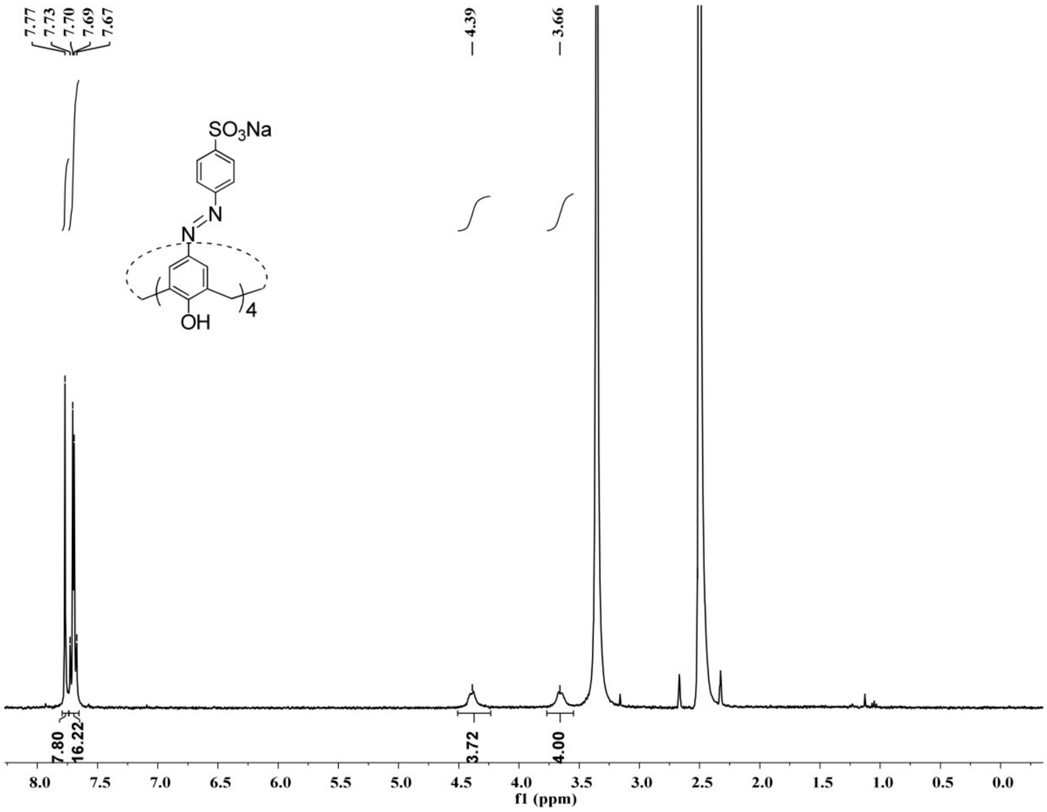

b

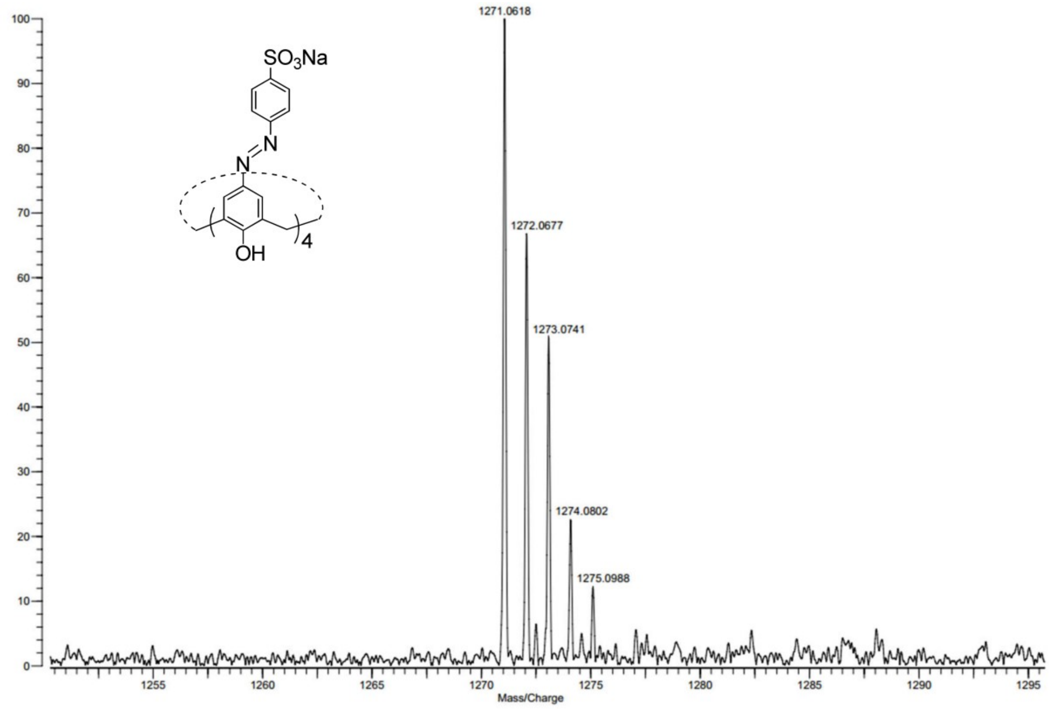

Figure S1. (a) ^{1}H NMR spectrum of SAC4A in DMSO-d_6, 400 MHz, 25 °C. (b) Mass spectrum (MALDI-TOF) of SAC4A.

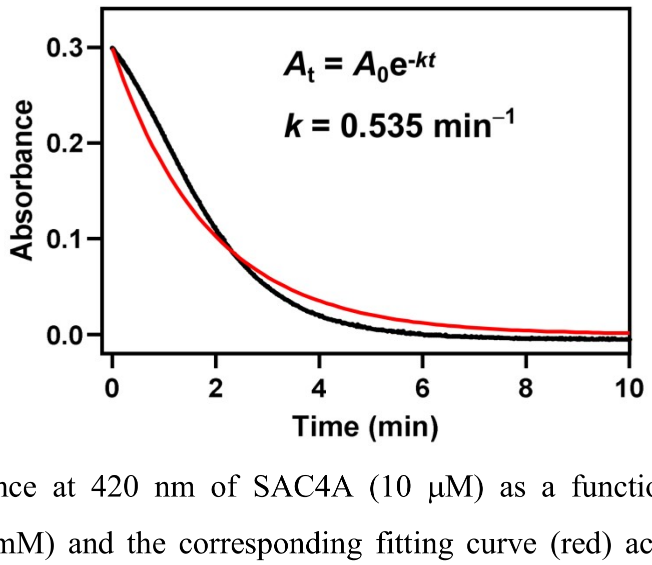

Figure S2. Absorbance at 420 nm of SAC4A (10 μM) as a function of time following addition of SDT (1 mM) and the corresponding fitting curve (red) according to quasi-first order reaction decay model. Experimental conditions: PBS buffer (10 mM, pH = 7.4), T = 25 °C.

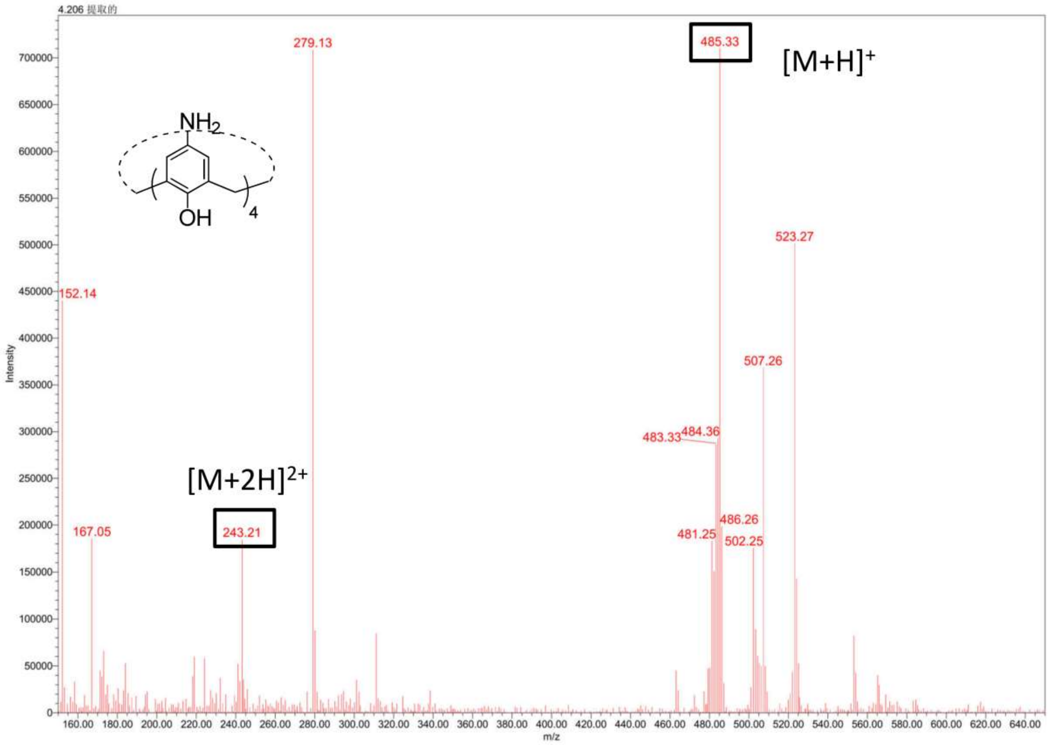

Figure S3. Mass spectrum of SAC4A after the incubation with SDT.

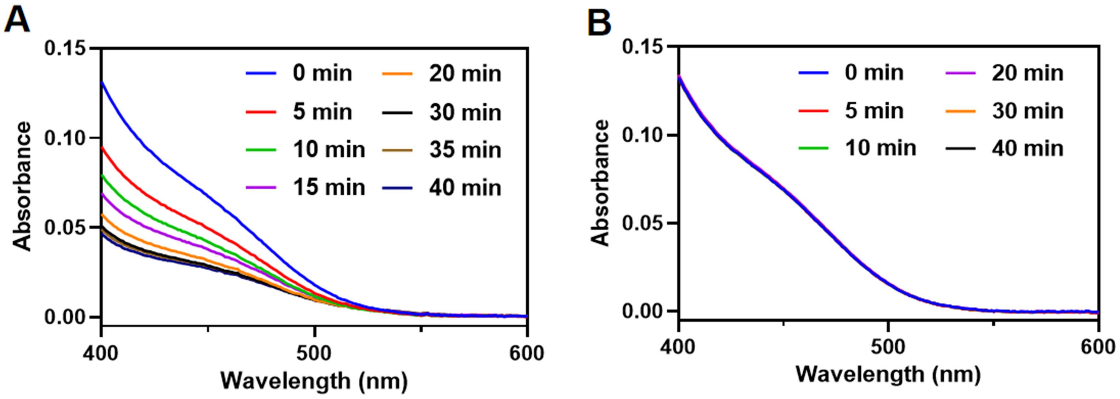

Figure S4. Absorption spectra of SAC4A (3 μM) against the time in the presence of DT-diaphorase (1 μM) and NADPH (50 μM) under hypoxic condition (A) or under normoxic condition (B). Experimental conditions: PBS buffer (10 mM, pH = 7.4), T = 37 °C.

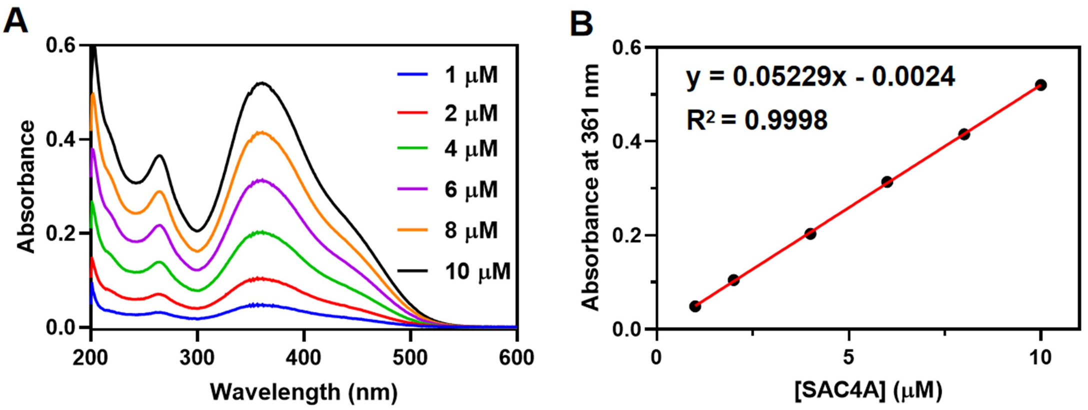

Figure S5. (A) Absorption spectra of SAC4A (1-10 μM). (B) The standard curve of SAC4A. Experimental conditions: PBS buffer (10 mM, pH = 7.4), 25 °C.

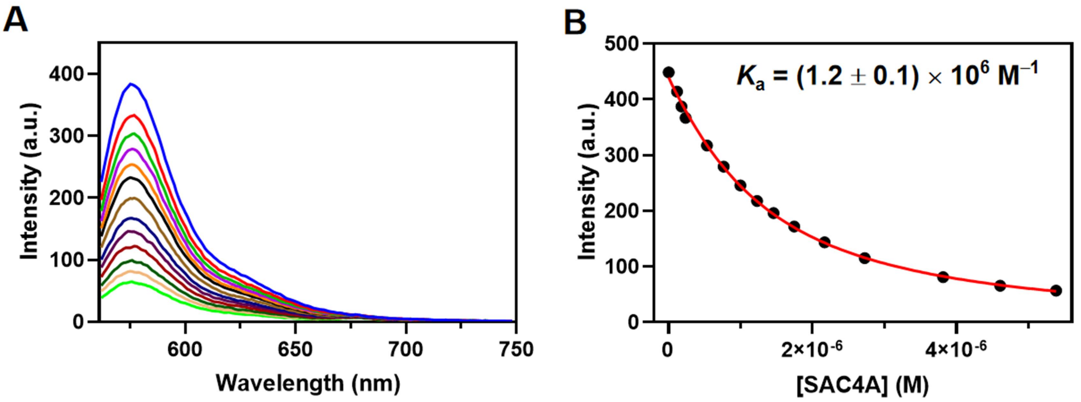

Figure S6. (A) Direct fluorescence titration of Rhodamine B (0.6 μM) with SAC4A (up to 5.4 μM) in PBS buffer (10 mM, pH = 7.4) at 25 °C, λ_{ex} = 554 nm. (B) The associated titration curve at λ_{em} = 575 nm was fitted according to the 1:1 binding stoichiometry.

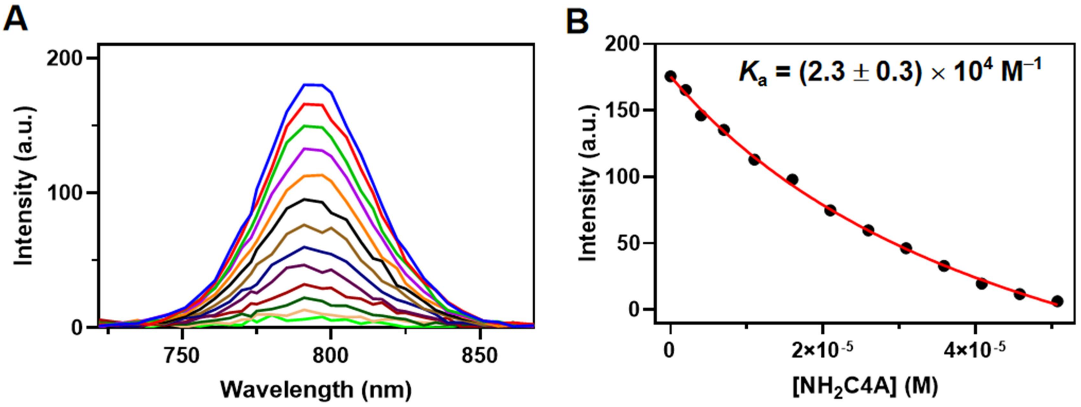

Figure S7. (A) Direct fluorescence titration of IR780 (2.0 μM) with NH_2C4A (up to 50.7 μM) in PBS buffer (10 mM, pH = 7.4) at 25 °C, λ_{ex} = 704 nm. (B) The associated titration curve at λ_{em} = 790 nm was fitted according to the 1:1 binding stoichiometry.

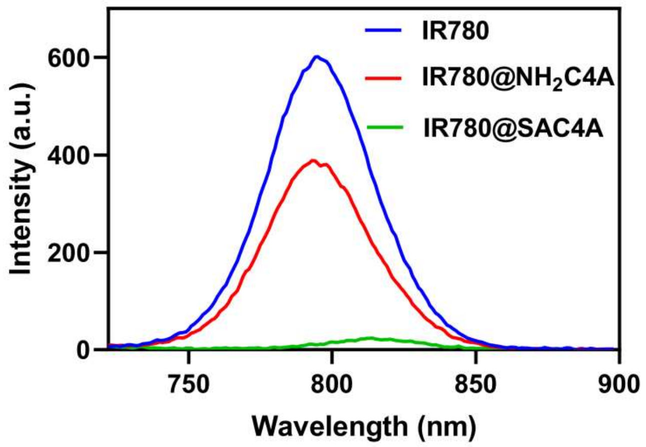

Figure S8. Fluorescence spectra of IR780 (10 μM), IR780@NH_2C4A (10/10 μM), and IR780@SAC4A (10 μM for IR780) in PBS buffer (10 mM, pH=7.4), 25 °C, λ_{ex} = 704 nm.

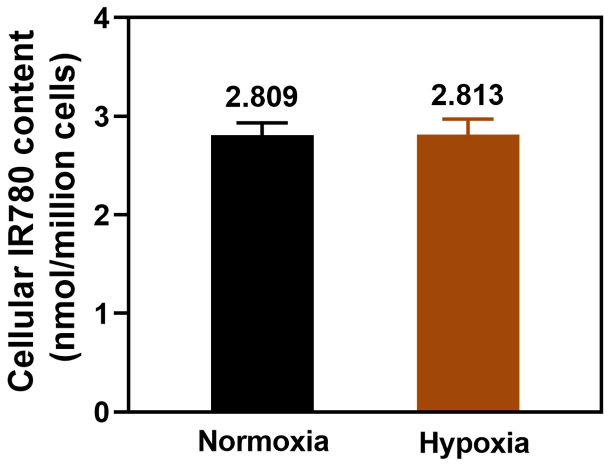

Figure S9. IR780 uptake in 4T1 cells after treatment with IR780@SAC4A under normoxic and hypoxic conditions for 6 h.

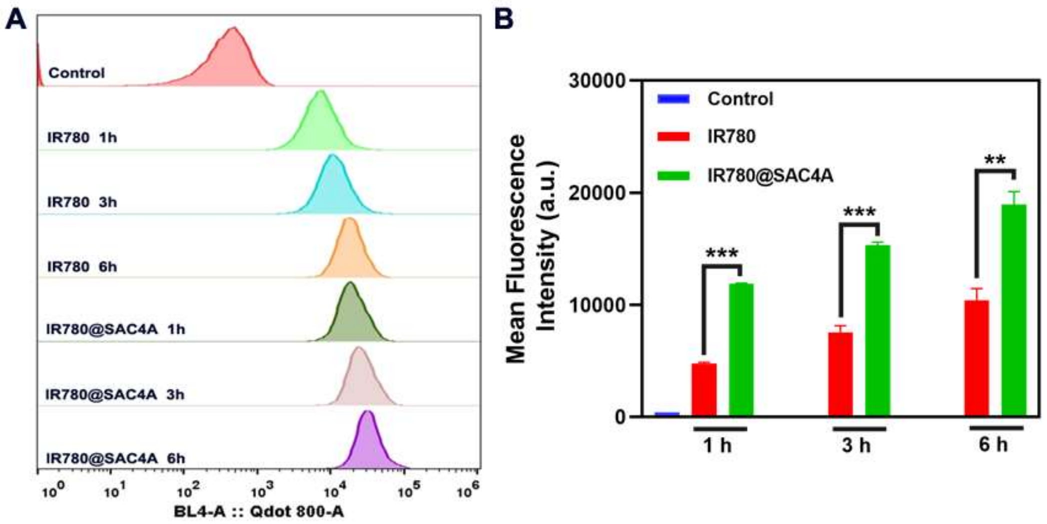

Figure S10. (A) Flow cytometry analyses of cellular IR780 in 4T1 cells after incubation with IR780 and IR780@SAC4A for 1, 3, and 6 h under hypoxic conditions. (B) Quantitative analyses of fluorescence intensities in cells after treated with IR780 and IR780@SAC4A for 1, 3, and 6 h under hypoxic conditions. The data are shown as mean ± SD (n = 3). P values were calculated by Student's t test: **$P < 0.01$, ***$P < 0.001$.

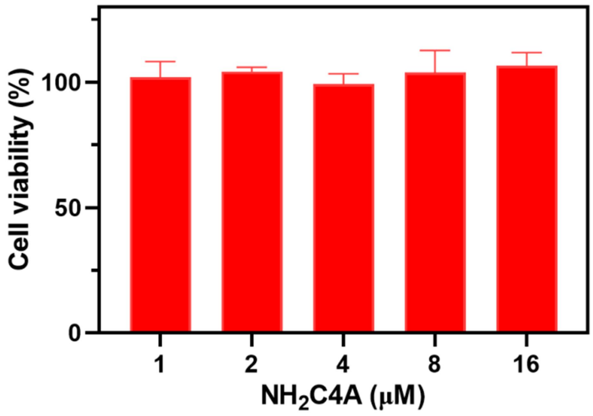

Figure S11. Cell viabilities of 4T1 cells treated with different concentrations of NH_2C4A for 24 h.

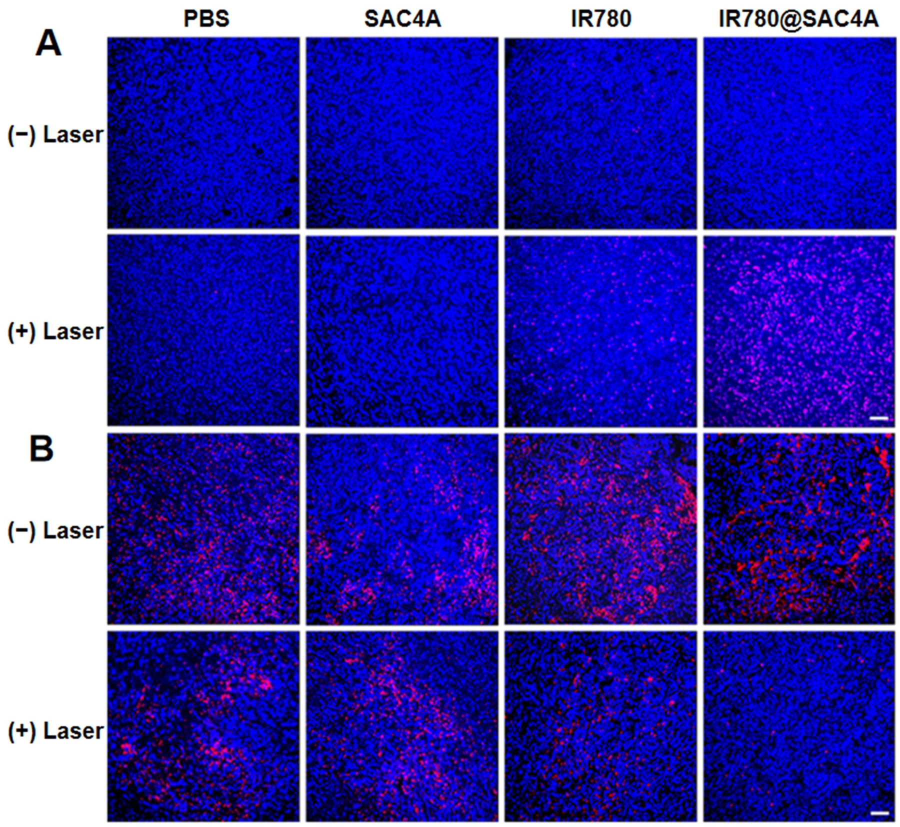

Figure S12. Immunofluorescence TUNEL (A) and Ki67 (B) staining of tumor slices in PBS±Laser, SAC4A±Laser, IR780±Laser, and IR780@SAC4A±Laser groups. Scale bar, 50 μm.

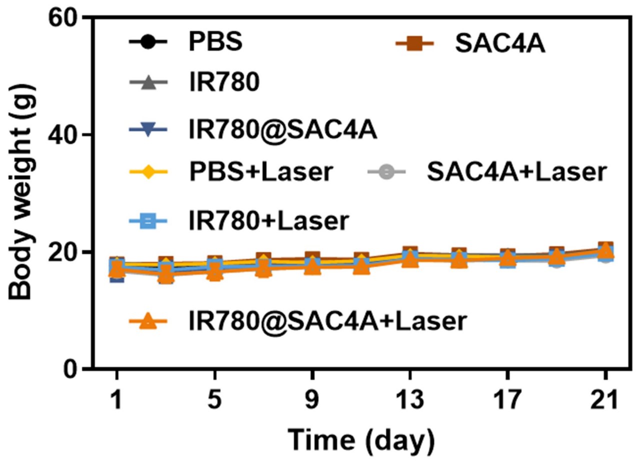

Figure S13. Body weight changes of tumor-bearing mice with PBS±Laser, SAC4A±Laser, IR780±Laser, and IR780@SAC4A±Laser treatments. The data are shown as mean ± SD (n = 5). Error bars could not be shown if less than 0.5.

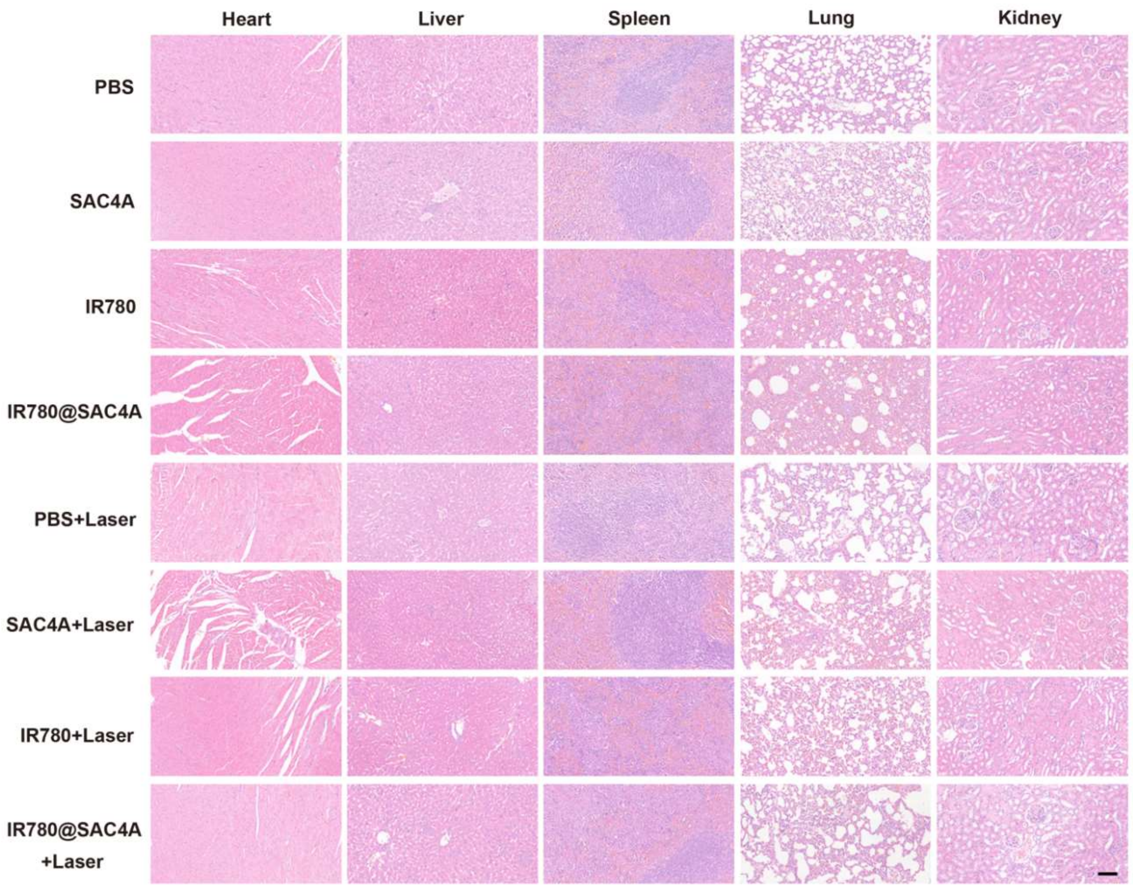

Figure S14. H&E staining of the major organs with PBS±Laser, SAC4A±Laser, IR780±Laser, and IR780@SAC4A±Laser treatments. Scale bar, 100 μm.

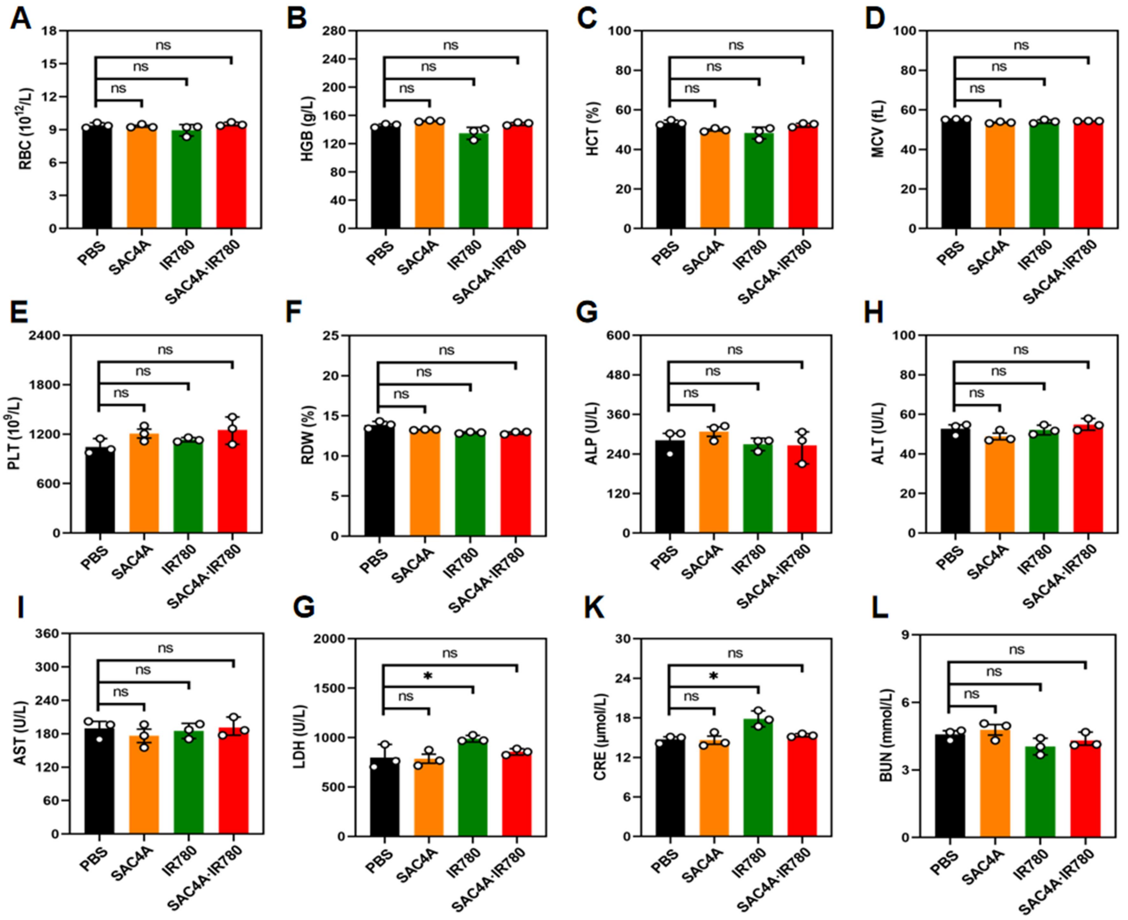

Figure S15. Blood biochemistry and hematology levels with PBS, SAC4A, IR780, and IR780@SAC4A treatments. The data are shown as mean ± SD (n = 3). P values were calculated by Student's t test: *$P < 0.05$. ns represents "no significant difference".

References

1. Zhang T-X, Zhang Z-Z, Yue Y-X, Hu X-Y, Huang F, Shi L, et al. A general hypoxia-responsive molecular container for tumor-targeted therapy. Adv Mater. 2020; 32: 1908435.
2. Guo D-S, Uzunova VD, Su X, Liu Y, Nau WM. Operational calixarene-based fluorescent sensing systems for choline and acetylcholine and their application to enzymatic reactions. Chem Sci. 2011; 2: 1722-1734.
3. Nau WM, Zhang X. An exceedingly long-lived fluorescent state as a distinct structural and dynamic probe for supramolecular association: an exploratory study of host−guest complexation by cyclodextrins. J Am Chem Soc. 1999; 121: 8022-8032.
4 Bakirci H, Nau WM. Fluorescence regeneration as a signaling principle for choline and

carnitine binding: a refined supramolecular sensor system based on a fluorescent azoalkane. Adv Funct Mater. 2006; 16: 237-242.

5. Hennig A, Bakirci H, Nau WM. Label-free continuous enzyme assays with macrocycle-fluorescent dye complexes. Nat Methods. 2007; 4: 629-632.
